# Identification and characterization of a novel botulinum neurotoxin

**DOI:** 10.1038/ncomms14130

**Published:** 2017-08-03

**Authors:** Sicai Zhang, Geoffrey Masuyer, Jie Zhang, Yi Shen, Daniel Lundin, Linda Henriksson, Shin-Ichiro Miyashita, Markel Martínez-Carranza, Min Dong, Pål Stenmark

**Affiliations:** 1 Department of Urology, Boston Children’s Hospital, Department of Microbiology and Immunobiology and Department of Surgery, Harvard Medical School, Boston, Massachusetts 02115, USA; 2 Department of Biochemistry and Biophysics, Stockholm University, SE-106 91 Stockholm, Sweden

## Abstract

Botulinum neurotoxins are known to have seven serotypes (BoNT/A–G). Here we report a new BoNT serotype, tentatively named BoNT/X, which has the lowest sequence identity with other BoNTs and is not recognized by antisera against known BoNTs. Similar to BoNT/B/D/F/G, BoNT/X cleaves vesicle-associated membrane proteins (VAMP) 1, 2 and 3, but at a novel site (Arg66-Ala67 in VAMP2). Remarkably, BoNT/X is the only toxin that also cleaves non-canonical substrates VAMP4, VAMP5 and Ykt6. To validate its activity, a small amount of full-length BoNT/X was assembled by linking two non-toxic fragments using a transpeptidase (sortase). Assembled BoNT/X cleaves VAMP2 and VAMP4 in cultured neurons and causes flaccid paralysis in mice. Thus, BoNT/X is a novel BoNT with a unique substrate profile. Its discovery posts a challenge to develop effective countermeasures, provides a novel tool for studying intracellular membrane trafficking, and presents a new potential therapeutic toxin for modulating secretions in cells.

## Abstract (web summary)

There are seven well-established types of Botulinum neurotoxins (BoNTs). Here the authors report the identification and characterization of a new type of BoNT—BoNT/X—which cleaves a different site on canonical BoNTs substrates and targets SNARE family members not cleaved by known BoNTs.

BoNTs are a family of bacterial toxins, classified as one of the six most dangerous potential bioterrorism agents (Category A and Tier 1 select agent in the United States)^1^. They are also widely used to treat a growing list of medical conditions^2^,^3^, including muscle spasms, chronic pain, overactive bladders, as well as having cosmetic applications. There are seven well-established serotypes of BoNTs (BoNT/A–G), traditionally defined based on a lack of cross-neutralization by different antisera raised against each toxin type. All BoNTs share the same structure and function^4^,^5^,^6^. They are composed of a light chain (LC, ∼50 kDa) and a heavy chain (HC, ∼100 kDa) connected by an inter-chain disulfide bond. The HC contains two sub-domains: the C-terminal H_C_ that mediates binding to receptors, and the N-terminal H_N_ that mediates translocation of the LC across endosomal membranes. The LC acts as a protease in neurons to cleave a set of proteins: BoNT/A, C and E cleave at distinct sites on a peripheral membrane protein known as SNAP-25; BoNT/B, D, F and G cleave at different sites on homologous vesicle proteins VAMP1, 2 and 3 (vesicle-associated membrane proteins); and BoNT/C also cleaves the plasma membrane protein syntaxin 1. These proteins are prototypes of the SNARE (soluble NSF attachment protein receptor) protein family, whose members mediate various membrane fusion events in eukaryotic cells^7^,^8^. Cleavage of any one of the three neuronal SNARE proteins blocks fusion of synaptic vesicles to plasma membranes, thus preventing neurotransmitter release from neurons.

Recognizing all distinct serotypes of BoNTs is essential for developing effective countermeasures against this family of toxins. BoNT/A and BoNT/B were first identified in 1919 by Georgina Burke^9^. The last of the seven serotypes, BoNT/G, was discovered in 1969 (ref. 10), and no new BoNT serotype has been found for the past four decades. Recent progress in genomic sequencing has revealed multiple subtypes (designated with Arabic numbers, for example, BoNT/A1), which can be recognized by the same antiserum but contain substantial sequence variations^11^,^12^,^13^. Furthermore, there are also multiple mosaic toxins. For instance, a ‘type H’ was reported in 2013 but was later designated as a mosaic toxin, as its LC shares ∼80% identity with the LC of a BoNT/F subtype, BoNT/F5, and its H_C_ shares ∼84% identity with the H_C_ of BoNT/A1 (refs 14, 15, 16, 17). Consistently, this toxin can be neutralized by antibodies against BoNT/A (ref. 16).

The genes encoding BoNTs can be on a plasmid, a phage, or the chromosome, indicating that these genes are mobile and capable of horizontal gene transfer^18^,^19^,^20^,^21^,^22^,^23^. Some strains contain two or even three different BoNT genes^15^,^24^,^25^. These strains are usually designated with a capital letter for the toxin type that is expressed at higher levels than the other one, followed with a lower case letter for the second toxin type (for example, BoNT/Af strain). In addition, it has also been reported that some BoNT/A strains contain a complete BoNT/B gene, but only BoNT/A is expressed^26^,^27^,^28^. Thus, the BoNT/B gene is considered a silent gene and the strains are known as BoNT/A(B) strains. A recent survey of infant botulism cases reported that ∼8% isolates are BoNT/A(B) strains^29^.

Here we searched published genomic sequences and identified a novel BoNT gene encoded on the chromosome of *Clostridium botulinum* strain 111. This strain was originally identified from an infant botulism patient in Japan in 1996 (ref. 30). The initial characterizations indicated that the toxicity of this strain is due to BoNT/B^30^. Later studies confirmed that this strain expresses a subtype of BoNT/B (BoNT/B2) encoded on a plasmid^31^,^32^. The sequence of this novel BoNT gene was deposited into the GenBank database in February 2015, as a part of the genomic sequence of *C. botulinum* 111. We characterized the protein encoded by this gene at functional levels and established it as a new BoNT serotype with a unique substrate profile.

## Results

### Searching genomic databases revealed a novel BoNT gene

**Figure 1:**
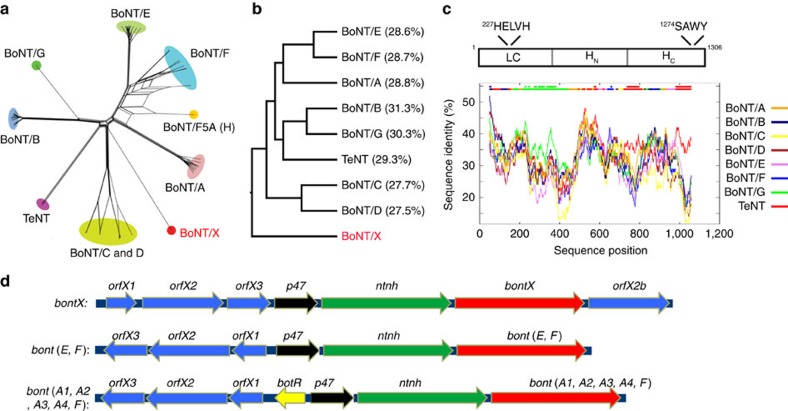
Identification of BoNT/X. (**a**) A phylogenetic split network covering all BoNT serotypes, subtypes, mosaic toxins and related tetanus neurotoxin (TeNT) illustrates their potential evolutionary relationships, as well as conflicts arising from e.g. chimerisms, based on their protein sequences. BoNT/X is highlighted in red. An enlarged version of this panel is shown in Supplementary Fig. 1, with the sequence access number for each toxin gene noted. (**b**) A phylogenic tree of the protein sequence alignment for BoNT/A-G, TeNT and BoNT/X, analysed by the ClustalW method. The percentages of sequence identity between each toxin and BoNT/X are noted. (**c**) Upper panel: a schematic drawing of the three domains of BoNT/X, with conserved protease motif in the LC and the ganglioside binding motif in the H_C_ noted. Lower panel: analysis using a sliding sequence comparison window demonstrated that the low similarity between BoNT/X and other BoNTs/TeNT is evenly distributed along the entire BoNT/X sequence. The *X* axis represents the query sequence position at the center of a 100-amino-acid moving sequence-comparison window. The *Y* axis shows the percentage of identity between that sequence window and each of the aligned background sequences. The two bars at the top of the graph illustrate the best matching sequence (lower bar) and whether the best match is significantly separated from the second-best match (upper bar). (**d**) A schematic drawing of the *orf* gene cluster that hosts the BoNT/X gene (upper panel), which has two distinct features compared with other known *orfX* clusters (middle and lower panels): (1) there is an additional *orfX2* protein (designated *orfX2b*) located next to the BoNT/X gene; (2) the reading frame of *orfX* genes has the same direction as the BoNT/X gene.

In an attempt to survey the evolutionary landscape of BoNTs, we performed iterative Hidden Markov model searches of the Uniprot sequence database. Our search identified all known BoNT subtypes and mosaic toxins, as well as the related tetanus neurotoxin (Fig. 1a; Supplementary Fig. 1). To our surprise, the search revealed a potentially new BoNT, tentatively designated BoNT/X (Fig. 1a, GenBank no.: BAQ12790.1), from the recently reported genomic sequence of *C. botulinum* strain 111. BoNT/X showed the least protein sequence identity with the other BoNTs in pairwise comparisons (Fig. 1b). Furthermore, the low sequence similarity is evenly distributed along the entire BoNT/X sequence (Fig. 1c), indicating that it is not a mosaic toxin. Despite this low sequence identity, the overall domain arrangement of BoNTs is conserved in BoNT/X (Fig. 1c), including a zinc-dependent protease motif HEXXH (residues 227–231, HELVH) in the LC (ref. 33), and a SXWY motif in the H_C_ (residues 1,274–1,277, SAWY), which recognizes the lipid receptor gangliosides^34^.

Similar to the other BoNTs, the BoNT/X gene is located in a gene cluster^23^. All seven established BoNTs are co-expressed with another 150 kDa protein known as NTNHA (non-toxic non-hemagglutinin protein), which forms a pH-dependent complex with BoNTs and protects them from proteases in the gastrointestinal tract^35^. The BoNT/X gene is also preceded by a potential NTNHA gene (Fig. 1d). Besides BoNT and NTNHA, a typical BoNT gene cluster contains genes encoding one of the two types of accessory proteins: (1) the HA cluster encoding three conserved proteins HA17, HA33 and HA70, which form a complex with BoNT/NTNHA and facilitate absorption of toxins across the intestinal epithelial barrier^36^,^37^,^38^; or (2) the OrfX cluster encoding conserved OrfX1, OrfX2, OrfX3 and P47 proteins with unknown function^23^. The BoNT/X gene is located in an OrfX gene cluster, as are BoNT/E, F and members of BoNT/A. Interestingly, the BoNT/X cluster has two unique features (Fig. 1d): (1) there is an additional OrfX2 gene that does not exist in any other BoNT clusters (we designated it OrfX2b); (2) the reading frame of OrfX genes is usually opposite to BoNT/NTNHA genes, but it has the same direction as the BoNT/X gene in the BoNT/X cluster (Fig. 1d). These findings suggest that BoNT/X is a unique branch of the BoNT family.

### The LC of BoNT/X cleaves VAMP2 at a novel site

**Figure 2:**
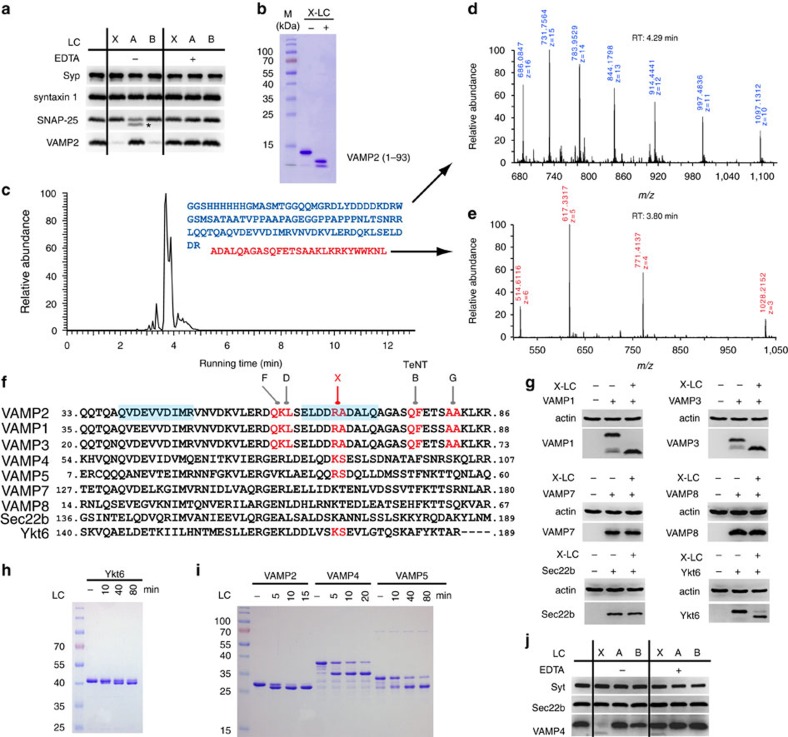
The LC of BoNT/X cleaves VAMPs at a unique site. (**a**) X-LC was incubated with BDE. Immunoblot analysis was carried out to detect syntaxin 1, SNAP-25 and VAMP2. Synaptophysin (Syp) served as a loading control. A-LC and B-LC were analysed in parallel. Cleavage of VAMP2 by B-LC results in loss of immunoblot signals, while cleavage of SNAP-25 by A-LC generates a smaller fragment (marked with an asterisk). EDTA blocked the activity of X-, A- and B-LCs. (**b**) VAMP2 (1–93) was incubated with X-LC. Samples were analysed by SDS–PAGE and Coomassie Blue staining. X-LC converted VAMP2 (1–93) into two smaller fragments. (**c**–**e**) VAMP2 (1–93) was incubated with X-LC. Samples were analysed by mass spectrometry (LC–MS/MS) to determine the molecular weight of cleaved fragments. Eluted peptide peaks from the HPLC column are plotted over running time (RT, *X* axis). The mass spectrometry data for the two cleavage products are colour-coded, with mass-to-charge ratio (*m*/*z*) noted. The molecular weight is deduced by multiplying *m* with *z*, followed by subtracting *z*. The protein sequences for the two cleavage products are colour-coded and listed in **c**. (**f**) Sequence alignment between VAMP family members, with the cleavage sites for BoNT/B, D, F, G and X marked in red, and the two SNARE motifs in blue shade. (**g**) HA-tagged VAMP1, 3, 7 and 8, and Myc-tagged Sec22b and Ykt6 were expressed in 293T cells via transient transfection. Cell lysates were incubated with X-LC and subjected to immunoblot analysis. Actin is a loading control. (**h**) GST-tagged Ykt6 was incubated with X-LC (100 nM). Samples were analysed by SDS–PAGE and Coomassie Blue staining. (**i**) GST-tagged VAMP2 (33–86), VAMP4 (1–115) and VAMP5 (1–70) were incubated with X-LC (100 nM). Samples were analysed by SDS–PAGE and Coomassie Blue staining. X-LC cleaved both VAMP4 and VAMP5. We note that VAMP5 protein contains a contaminant band that runs close to the cleavage product. (**j**) Experiments were carried out as described in **a**, except that VAMP4 and Sec22b were detected. Synaptotagmin I (Syt I) is a loading control. X-LC cleaved native VAMP4 in BDE. One of two (**b**,**g**,**j**) or three (**a**,**h**,**i**) independent experiments is shown.

To characterize BoNT/X, we first focused on its LC (X-LC, residues 1–439) and produced it as a His6-tagged protein in *Escherichia coli.* LCs of BoNT/A (A-LC) and BoNT/B (B-LC) were produced and assayed in parallel as controls. Incubation of X-LC with rat brain detergent extracts (BDE) did not affect syntaxin 1 or SNAP-25, but abolished VAMP2 immunoblot signals (Fig. 2a). LCs of BoNTs are zinc-dependent proteases^33^. As expected, EDTA prevented cleavage of SNARE proteins by X-, A- and B-LCs (Fig. 2a). Furthermore, incubation of X-LC with the purified recombinant cytosolic domain of VAMP2 (residues 1–93) converted VAMP2 into two lower-molecular-weight bands (Fig. 2b), confirming that X-LC cleaves VAMP2.

To identify the cleavage site, we analysed the VAMP2 (1–93) protein, with or without pre-incubation with X-LC, by liquid chromatography–tandem mass spectrometry (LC–MS/MS, Fig. 2c–e). A single dominant peptide peak appeared after incubation with X-LC (Fig. 2c,e; Supplementary Fig. 2). Its molecular weight is 3,081.7, which fits only the peptide sequence of A67-L93 of VAMP2 (Fig. 2c,e). Consistently, another fragment from the beginning of the His6-tag to residue R66 of VAMP2 was also detected (Fig. 2d). To further confirm this finding, we repeated the assay with a different VAMP2 fragment: glutathione *S*-transferase (GST) tagged VAMP2 (33–86) (Supplementary Fig. 3). Incubation with X-LC generated a single dominant peptide peak with a molecular weight of 2,063.1, which fits only A67-R86 of VAMP2 (Supplementary Fig. 3). Together, these results demonstrate that X-LC has a single cleavage site on VAMP2 between R66 and A67.

R66-A67 is a novel cleavage site on VAMP2, distinct from all established target sites of BoNTs (Fig. 2f). It is also the only BoNT cleavage site located within a region previously known as the SNARE motif (Fig. 2f, shaded regions)^39^. The VAMP protein family includes VAMP1, 2, 3, 4, 5, 7 and 8, as well as related Sec22b and Ykt6. R66-A67 is conserved in VAMP1 and 3, which are highly homologous to VAMP2. To validate the specificity of X-LC, we expressed HA-tagged VAMP1, 3, 7, 8 and Myc-tagged Sec22b and Ykt6 in HEK293 cells via transient transfection. Cell lysates were incubated with X-LC. Both VAMP1 and 3 were cleaved by X-LC, whereas VAMP7, VAMP8 and Sec22b were resistant to X-LC (Fig. 2g).

### BoNT/X cleaves VAMP4, VAMP5 and Ykt6

Unexpectedly, Ykt6 was also cleaved by X-LC (Fig. 2g). This finding was confirmed using a purified GST-tagged Ykt6 fragment, which shifted to a lower-molecular-weight band after incubation with X-LC (Fig. 2h). The cleavage site was determined to be K173-S174 by mass spectrometry analysis of the intact Ykt6 versus Ykt6 cleaved by X-LC (Supplementary Fig. 4). This site is homologous to the cleavage site of BoNT/X on VAMP2 (Fig. 2f). Among VAMP family of proteins, VAMP4 contains the same pair of residues (K87-S88) at this site as Ykt6. We found that X-LC cleaved both purified GST-tagged cytoplasmic domain of VAMP4 (Fig. 2i), as well as native VAMP4 in BDE (Fig. 2j). As a control, Sec22b was not cleaved by X-LC in BDE. In addition, the GST-tagged cytoplasmic domain of VAMP5 was also cleaved (Fig. 2i). The cleavage sites were determined by mass spectrometry analysis to be K87-S88 in VAMP4 and R40-S41 in VAMP5 (Supplementary Fig. 5). Both sites are homologous to the cleavage site of BoNT/X on VAMP2 (Fig. 2f), demonstrating that the location of the cleavage site is conserved across different VAMPs. The ability of X-LC to cleave VAMP4, VAMP5 and Ykt6 is highly unusual, as their sequences are substantially different from VAMP1/2/3. BoNT/X is the first and the only BoNT known that can cleave VAMPs beyond the canonical targets VAMP1, 2 and 3 (ref. 40).

### Proteolytic activation of BoNT/X

**Figure 3:**
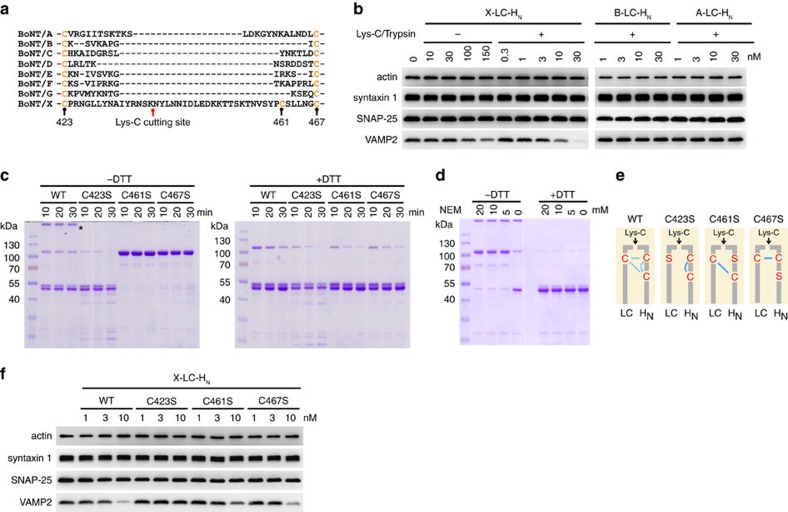
Proteolytic activation and inter-chain disulfide bond in BoNT/X. (**a**) Sequence alignment of the linker between the LC and HC of the seven established BoNTs plus BoNT/X. The Lys-C cutting site was identified by mass spectrometry analysis (see Method and Supplementary Data 1). (**b**) Cultured rat cortical neurons were exposed to X-LC-H_N_ for 12 h. Cell lysates were harvested and immunoblot analysis carried out to examine syntaxin 1, SNAP-25 and VAMP2. Actin is a loading control. Trypsin-activated A-LC-H_N_ and B-LC-H_N_ were analysed in parallel. X-LC-H_N_ entered neurons and cleaved VAMP2. X-LC-H_N_ activated by Lys-C showed a greater potency than non-activated X-LC-H_N_. X-LC-H_N_ was more potent than B-LC-H_N_ and A-LC-H_N_, neither of which cleaved their substrates. (**c**) WT and mutant X-LC-H_N_ were activated by Lys-C and analysed by SDS–PAGE and Coomassie Blue staining, with or without DTT. C461S and C467S mutants showed as a single band at ∼100 kDa without DTT, and separated into two ∼50 kDa bands with DTT. A portion of WT X-LC-H_N_ formed aggregates, marked by an asterisk, which disappeared with DTT. The majority of activated WT X-LC-H_N_ separated into two ∼50 kDa bands without DTT. This is due to disulfide bond shuffling as described in the following panel. (**d**) Lys-C-activated WT X-LC-H_N_ was incubated with NEM to block disulfide bond shuffling. Samples were then analysed by SDS–PAGE and Coomassie Blue staining. A majority of WT X-LC-H_N_ exists as a single band at ∼100 kDa without DTT after NEM treatment, indicating that native WT X-LC-H_N_ contains an inter-chain disulfide bond. (**e**) Schematic drawings of the disulfide bond in WT and three cysteine mutants of BoNT/X. (**f**) Experiments were carried out as described in **b**, except that neurons were exposed to WT or X-LC-H_N_ mutants. C423S mutation abolished the activity of X-LC-H_N_, whereas mutating C461 or C467 did not affect the activity of X-LC-H_N_. These results confirmed that the inter-chain disulfide bond is essential for the activity of X-LC-H_N_, and this inter-chain disulfide bond can be formed via either C423-C461 or C423-C467. One of two (**b**) or three (**b**,**c**,**f**) independent experiments is shown.

We next examined the linker region between the LC and the HC, which must be cleaved by bacterial or host proteases to convert the toxin to an ‘active’ di-chain form. We produced a recombinant X-LC-H_N_ fragment (residues 1–891) in *E. coli* and subjected it to limited proteolysis by endoproteinase Lys-C. Samples were analysed using Tandem Mass Tag (TMT) labelling and tandem mass spectrometry. TMT labels free N-termini (and lysines). Limited proteolysis by Lys-C produced one new free N terminus mapped to residue N439 in the linker region (Fig. 3a; Supplementary Data 1), confirming that the linker region is susceptible to proteases.

We then examined whether proteolytic activation enhances the potency of BoNT/X. It has been shown that incubation of high concentrations of LC-H_N_ of BoNTs with cultured neurons results in entry of LC-H_N_, likely through non-specific uptake into neurons^41^. Similarly, X-LC-H_N_ entered cultured rat cortical neurons and cleaved VAMP2 in a concentration-dependent manner (Fig. 3b). Activation by Lys-C increased the potency of X-LC-H_N_: 10 nM activated X-LC-H_N_ cleaved similar levels of VAMP2 as 150 nM intact X-LC-H_N_ (Fig. 3b). Activated X-LC-H_N_ appears to be more potent than activated LC-H_N_ of BoNT/A (A-LC-H_N_) and BoNT/B (B-LC-H_N_), which did not show any detectable cleavage of their substrates under the same assay conditions (Fig. 3b).

### The inter-chain disulfide bond in BoNT/X

Like other BoNTs, the linker region of BoNT/X contains two conserved cysteines, but there is also an additional cysteine (C461) unique to BoNT/X (Fig. 3a). To determine the cysteine residues that form the essential inter-chain disulfide bond, we generated three X-LC-H_N_ mutants, each with one of the three cysteine residues mutated (C423S, C461S and C467S). These mutants, as well as wild-type (WT) X-LC-H_N_, were subjected to limited proteolysis with Lys-C and then analysed via SDS–PAGE and Coomassie Blue staining, with or without the reducing agent dithiothreitol (DTT; Fig. 3c). Mutating the only cysteine on the LC (C423S) is expected to abolish the inter-chain disulfide bond. Consistently, C423S mutant separated into two ∼50 kDa bands without DTT. In contrast, both C461S and C467S mutants showed as a single band at 100 kDa in the absence of DTT and separated into two ∼50 kDa bands in the presence of DTT. These results suggested that C423 on the LC can form the inter-chain disulfide bond with either C461 or C467 on the HC. We also found that Lys-C treatment degraded a significant portion of C423S mutant as compared with C461S or C467S mutants (Fig. 3c, +DTT), suggesting that losing the inter-chain disulfide bond makes the molecule more susceptible to proteases. We noticed that a portion of WT X-LC-H_N_ formed aggregates at the top of the SDS–PAGE gel (Fig. 3c, marked by an asterisk). These aggregates disappeared in the presence of DTT. C423/C461/C467 are the only three cysteines in the X-LC-H_N_; mutating any one of them abolished formation of aggregates (Fig. 3c, −DTT), suggesting that these aggregates are formed by inter-molecular disulfide bonds due to the existence of an extra cysteine in the linker region.

Interestingly, the majority of activated WT X-LC-H_N_ separated into two ∼50 kDa bands without DTT (Fig. 3c), which is similar to C423S mutant. On the other hand, WT X-LC-H_N_ did not show increased degradation by Lys-C compared with C423S mutant (Fig. 3c, +DTT). One possible explanation is that WT X-LC-H_N_ contains an inter-chain disulfide bond under native conditions, but this bond can rearrange to intra-chain C461–C467 pair under denaturing conditions in the SDS buffer. This phenomenon is known as disulfide bond shuffling, which often occurs among adjacent cysteines. To test this hypothesis, we utilized an alkylating reagent, *N-*Ethylmaleimide (NEM), which permanently blocks free cysteines and prevents disulfide bond shuffling. As shown in Fig. 3d, WT X-LC-H_N_ pretreated with NEM showed as a single band at 100 kDa in the absence of DTT, and separated into two ∼50 kDa bands in the presence of DTT. These results confirm that WT X-LC-H_N_ contains mainly an inter-chain disulfide bond, but it is susceptible to disulfide bond shuffling due to an extra cysteine in the linker region (Fig. 3e).

We further examined the activity of the three X-LC-H_N_ cysteine mutants on cultured neurons. As expected, C423S mutant was inactive, whereas C461S and C467S mutants both showed similar levels of activity as WT X-LC-H_N_ (Fig. 3f). These results confirm that the inter-chain disulfide bond is critical for the activity of BoNT/X.

### Generating full-length BoNT/X via sortase-mediated ligation

**Figure 4:**
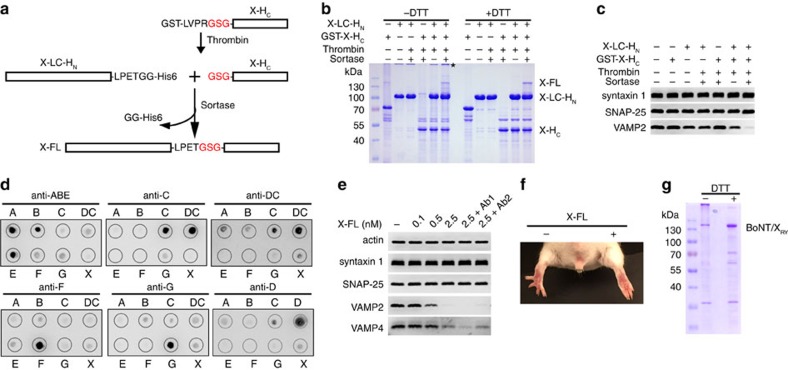
Full-length BoNT/X is active on cultured neurons and *in vivo* in mice. (**a**) A schematic drawing of the sortase ligation method. (**b**) Sortase ligation reaction mixtures were analysed by SDS–PAGE and Coomassie Blue staining. The asterisk marks the proteins aggregates due to inter-molecular disulfide bonds. Full-length BoNT/X (X-FL) appeared only in the sortase ligation mixture. (**c**) Neurons exposed to the sortase ligation mixture (15 μl) or control mixtures for 12 h in culture medium. Cell lysates were analysed by immunoblot. The mixture containing both X-LC-H_N_ and X-H_C_ (but not sortase) cleaved slightly more VAMP2 than X-LC-H_N_ alone. Ligating X-LC-H_N_ and X-H_C_ by sortase further enhanced cleavage of VAMP2, demonstrating that ligated X-FL is functional on neurons. (**d**) BoNT/A-G, BoNT/DC and BoNT/X were subjected to the dot blot assay, using four horse antisera (trivalent anti-BoNT/A, B and E, anti-BoNT/C, anti-BoNT/DC and anti-BoNT/F), as well as two goat antisera (anti-BoNT/G and anti-BoNT/D). BoNT/X is composed of X-LC-H_N_ and X-H_C_ at 1:1 molar ratio. These antisera recognized their corresponding target toxins, yet none recognized BoNT/X. The antisera against BoNT/DC and BoNT/C cross-react, as these two toxins share a high degree of similarity within their H_C_ domains. (**e**) Cultured rat cortical neurons were exposed to ligated X-FL in culture medium for 12 h, with or without two combinations of anti-sera. Ab1: trivalent anti-BoNT/A/B/E, anti-BoNT/C and anti-BoNT/F. Ab2: anti-BoNT/G and anti-BoNT/D. The trivalent anti-BoNT/A/B/E was used at 1:50 dilution. All other anti-sera were used at 1:100 dilution. None of the antisera affected the cleavage of VAMP2 and VAMP4 by X-FL. The specificity and potency of these antisera were validated for their ability to neutralize target serotypes in the same assay as described in Supplementary Fig. 7. (**f**) X-FL linked by sortase reaction (0.5 μg) was injected into the gastrocnemius muscles of the right hind limb of mice (*n*=4). The injected limb developed typical flaccid paralysis, and the toes failed to spread within 12 h. The left limb was not injected with toxins, serving as a control. (**g**) Full-length inactive form of BoNT/X (BoNT/X_RY_) was purified as a His6-tagged recombinant protein in *E. coli*. Further purified BoNT/X_RY_ is shown in Supplementary Fig. 8b. One of two (**e**) or three (**c**,**d**) independent experiments is shown.

We then sought to determine whether full-length BoNT/X is a functional toxin. As no antisera against BoNT/X are available, we decided to avoid generating the full-length active toxin gene. Instead, we developed an approach to generate a limited amount of full-length BoNTs in test tubes by enzymatic ligation of two non-toxic fragments of BoNTs. This method utilizes a transpeptidase known as sortase^42^,^43^, which recognizes the peptide motif LPXTG, cleaves between T-G, and concurrently forms a new peptide bond with other proteins/peptides containing N-terminal glycine (Fig. 4a). We produced two non-toxic fragments of BoNT/X: (1) LC-H_N_ with a LPETGG motif and a His6-tag fused to the C terminus; and (2) the H_C_ of BoNT/X (X-H_C_) with a GST tag, thrombin cleavage site, and an additional glycine residue at its N terminus. Cutting by thrombin releases X-H_C_ with a free glycine at its N terminus. Incubation of these two fragments with sortase generated a small amount of ∼150 kD full-length BoNT/X (X-FL, Fig. 4a,b). We note that X-H_C_ showed poor solubility and a strong tendency towards aggregation, which might be the reason for the low ligation efficiency (Fig. 4b). In contrast, ligation of X-LC-H_N_ with the H_C_ of BoNT/A (A-H_C_) achieved a better efficiency, with the majority of X-LC-H_N_ ligated into a XA chimeric toxin (Supplementary Fig. 6a). To ensure biosafety, the amount of precursor fragments in the reaction is strictly limited to generate the minimum amount of ligated toxin necessary for functional assays.

We first analysed the activity of ligated BoNT/X using cultured rat cortical neurons. Neurons were exposed to the sortase ligation mixture and control mixtures in culture medium. As shown in Fig. 4c, X-LC-H_N_ alone cleaved some VAMP2 due to its high concentration in the reaction mixture. Mixing X-H_C_ with X-LC-H_N_ without sortase slightly enhanced cleavage of VAMP2 compared with X-LC-H_N_ alone, suggesting that X-H_C_ might be associated with X-LC-H_N_ via non-covalent interactions. This interaction appears to be specific, as mixing A-H_C_ with X-LC-H_N_ did not enhance cleavage of VAMP2 in neurons (Supplementary Fig. 6b). Ligating X-LC-H_N_ with X-H_C_ by sortase clearly enhanced cleavage of VAMP2 compared with the mixture of X-LC-H_N_ and X-H_C_ without sortase (Fig. 4c). These results demonstrated that the X-H_C_ is functional for targeting cells and that ligated full-length BoNT/X entered neurons and cleaved VAMP2. Similarly, ligated XA also entered neurons and cleaved VAMP2 (Supplementary Fig. 6b).

### BoNT/X was not recognized by antisera against known BoNTs

We next carried out dot blot assays using antisera raised against known BoNTs, including all seven serotypes as well as one mosaic toxin (BoNT/DC), to confirm that BoNT/X is serologically unique. Four horse antisera were utilized (trivalent anti-BoNT/A, B and E, anti-BoNT/C, anti-BoNT/DC, and anti-BoNT/F) as well as two goat antisera (anti-BoNT/G and anti-BoNT/D). The specificity and potency of these antisera were first validated by analysing their ability to neutralize BoNTs on cultured neurons. As expected, all antisera neutralized their target BoNTs, without affecting the activity of a different serotype (Supplementary Fig. 7). We found that these antisera recognized their corresponding BoNTs in the dot blot assay, yet none recognized BoNT/X (Fig. 4d).

We further analysed whether the toxicity of BoNT/X on neurons can be neutralized by these antisera. X-FL generated by sortase-mediated ligation was first activated with limited proteolysis using trypsin. We used trypsin to activate X-FL instead of Lys-C for functional assays, as trypsin allows us to stop proteolysis using trypsin inhibitors. Activated X-FL entered cultured rat cortical neurons and cleaved both VAMP2 and VAMP4 in a concentration-dependent manner (Fig. 4e). Combinations of antisera against known BoNTs (Ab1 (horse antisera): trivalent anti-BoNT/A, B and E, anti-BoNT/C, and anti-BoNT/F; Ab2 (goat antisera): anti-BoNT/G and anti-BoNT/D) did not affect the activity of ligated X-FL, as evidenced by similar degrees of VAMP2 and VAMP4 cleavage in the presence of these antisera (Fig. 4e). These results confirmed that BoNT/X is a new BoNT serotype.

### BoNT/X induced flaccid paralysis *in vivo* in mice

We next sought to determine whether BoNT/X is active *in vivo* using a well-established non-lethal assay in mice, known as the Digit Abduction Score (DAS) assay, which measures local muscle paralysis following injection of BoNTs into mouse hind limb muscles^44^. BoNTs cause flaccid paralysis of limb muscles, which is manifested as the failure to spread the toes in response to a startle stimulus. We injected ligated X-FL (0.5 μg, activated by trypsin treatment) into the gastrocnemius muscles of the right hind limb in mice, which induced typical flaccid paralysis and the failure of toes to spread (Fig. 4f), indicating that BoNT/X is capable of causing flaccid paralysis *in vivo*. We note that the potency of ligated X-FL appears to be much lower than other BoNTs in this assay. To further confirm the low toxicity of ligated X-FL, we injected mice with 1 μg of ligated X-FL intraperitoneally (*n*=3). No mice showed any systemic effects and all survived at this dose. Thus, ligated X-FL has a rather low toxicity *in vivo* in mice compared with other native BoNTs, which usually have lethal doses at low picogram levels per mouse.

### Full-length inactive BoNT/X

Finally, we developed an inactive mutant of BoNT/X as a potential reagent for generating neutralizing antibodies. Mutations at two residues (R362A/Y365F) in BoNT/A inactivate the protease activity of the LC and abolish the toxicity of BoNT/A *in vivo*^45^,^46^. These two residues are conserved in BoNT/X. We introduced the corresponding mutations (R360A/Y363F) in BoNT/X and generated a full-length inactive form, designated as BoNT/X_RY_. As shown in Fig. 4g, BoNT/X_RY_ was purified as a His6-tagged protein in *E. coli*, and it had no activity on cultured neurons (Supplementary Fig. 8a). Furthermore, intraperitoneal injection of mice with 30 μg BoNT/X_RY_ (activated by trypsin treatment) did not cause any adverse effects (*n*=5), demonstrating that it is not toxic *in vivo*. A substantial portion of BoNT/X_RY_ formed aggregates at the top of the SDS–PAGE gel (Fig. 4g). Adding DTT reduced these aggregates to monomeric BoNT/X_RY_ (Fig. 4g). Thus, full-length BoNT/X is susceptible to forming inter-molecular disulfide bonds. Nevertheless, the monomeric form of BoNT/X can be purified and is stable in solution (Fig. 4g). Furthermore, we developed a scale-up purification protocol, which generated BoNT/X_RY_ with a yield of ∼3 mg per liter of culture and ∼90% purity (Supplementary Fig. 8b). Highly purified BoNT/X_RY_ remained stable in solution up to 10 mg ml^−1^ in the presence of reducing agent. This atoxic BoNT/X_RY_ will be a valuable reagent for generating neutralizing antibodies.

## Discussion

BoNT/X is the first serotype of BoNTs identified by genomic sequencing and bioinformatics approaches. It remains unknown whether BoNT/X is ever produced in *C. botulinum* strain 111. BoNT/X could be a silent gene, or it may not be expressed at detectable toxicity levels under culture conditions in the lab. Thus, this toxin was revealed only by sequencing *C. botulinum* 111. This illustrates the importance of genomic sequencing and bioinformatics approaches for understanding microbial virulence factors. Whether BoNT/X could be expressed and exhibit toxicity under certain environmental conditions remains an intriguing question.

A remarkable feature of BoNT/X is its unique ability to cleave VAMP4 and Ykt6. VAMP4 is widely expressed and is known to mediate vesicle fusion between the trans-Golgi network (TGN) and endosomes, as well as homotypic fusion of endosomes^47^,^48^. Ykt6 is an atypical SNARE without a transmembrane domain^49^. It is anchored to membranes via lipidation, which allows dynamic regulation of its membrane association. Ykt6 is an essential protein in yeast and implicated in multiple membrane fusion events including ER-Golgi, intra-Golgi, endosome-Golgi-vacuolar, and autophagosome formation. Its function in mammalian cells remains to be established. BoNT/X is the first and only BoNT to cleave these SNAREs that mediate various intracellular membrane-trafficking events.

Interestingly, both VAMP4 and Ykt6 are enriched in neurons. Recent studies suggested that VAMP4 contributes to asynchronous synaptic vesicle exocytosis, enlargeosome exocytosis and activity-dependent bulk endocytosis (ADBE) in neurons^50^,^51^,^52^. The role of Ykt6 in neurons remains to be established, but it has been shown to suppress the toxicity of α-synuclein in Parkinson’s disease models^53^,^54^. The other substrate of BoNT/X, VAMP5, is mainly expressed in muscle cells and its function remains to be established^55^. BoNT/X will be a useful tool to investigate the function of VAMP4, Ykt6 and VAMP5, as well as related membrane trafficking events. In addition, because VAMP4 has been implicated in granule release in immune cells^56^, BoNT/X may have the potential to modulate inflammatory secretion in immune cells.

The X-LC-H_N_ fragment showed a higher level of activity in neurons than either A-LC-H_N_ or B-LC-H_N_, suggesting that its membrane translocation and/or protease activity might be more efficient than the corresponding fragments in BoNT/A and BoNT/B. X-H_C_ is functional for targeting cells, as its presence enhanced cleavage of VAMP2 in neurons over LC-H_N_ alone (Fig. 4c). When present without the translocation and LC domains, X-H_C_ is prone to aggregation. This solubility issue is likely due to separation of X-H_C_ from X-LC-H_N_, as full-length BoNT/X_RY_ remains stable at high concentrations. The X-FL generated by sortase-mediated ligation has a rather low toxicity *in vivo* in mice. It remains unknown whether this low *in vivo* toxicity was intrinsic to BoNT/X. It is also possible that the sortase linking method resulted in an attenuated toxin, as the H_C_ and LC-H_N_ folded separately and there is an additional linker between the H_N_ and H_C_ in ligated toxins. Nevertheless, X-FL produced by sortase-mediated ligation is active on neurons and induced typical flaccid paralysis when injected locally in mice, demonstrating that BoNT/X is a functional toxin (Fig. 4f). It will be necessary to produce native BoNT/X to characterize its *in vivo* potency and determine its biosafety risk. It will be important to generate neutralizing antisera against BoNT/X before producing any native toxin.

Introducing a full-length active toxin gene into any expression system/organism is always a significant biosafety concern. Sortase-mediated ligation assembles a small quantity of full-length toxin from two complementary and non-toxic fragments expressed and purified individually. The amount of the precursor fragments in the reaction can be strictly controlled, so the amount of ligated toxin is precisely limited to ensure biosafety. The ligated toxin should possess the same mode of action as native toxins, but may exhibit lower toxicity *in vivo*, possibly due to the addition of the sortase linker and/or reduced compactness of the molecule. Thus, the ligated toxin could be used for functional studies, but may not be suitable for estimating the lethal dose of native toxin. On the other hand, this reduced toxicity from the sortase linking method could be an advantage to create attenuated toxins for research use. It might be also possible to deliberately attenuate the toxicity of ligated toxins *in vivo* by including additional linkers, such as peptide sequences that are sensitive to serum proteases, to further mitigate biosafety concerns.

## Methods

### Materials

Mouse monoclonal antibodies for syntaxin 1 (HPC-1, dilution 1:5,000), SNAP-25 (C171.2, 1:5,000), VAMP2 (C169.1, 1:2,000), Syp (Cl7.2, 1:2,000) and Syt I (mAB48, 1:1,000) were generously provided by E. Chapman (Madison, WI, USA). Most are available from Synaptic Systems (Goettingen, Germany), with the exception of Syt I (mAB48), which is available from the Developmental Studies Hybridoma Bank. Rabbit polyclonal antibodies against VAMP4 (Cat. no. 136002, 1:1,000) and Sec22b (Cat. no. 186003, 1:1,000) were purchased from Synaptic Systems. The following mouse monoclonal antibodies were purchased from the indicated vendors: actin (Sigma, AC-15, 1:2,000); anti-HA (Covance, 16B12, 1:2,000); anti-Myc (Millipore, 9E10, 1:1,000). Equine polyclonal antisera against BoNT/A/B/E, BoNT/C, BoNT/DC, BoNT/F and goat polyclonal antisera against BoNT/G were generously provided by S. Sharma (FDA). Goat polyclonal antibody against BoNT/D was purchased from Fisher Scientific (NB10062469). BoNTs were purchased from Metabiologics (Madison, WI, USA). Antibody validation is available on the manufacturers’ websites. 293T (#CRL-3216) cells were originally obtained from ATCC, which were negative for mycoplasma contamination but have not been authenticated.

### cDNA and constructs

The cDNAs encoding X-LC (residues 1–439), X-H_C_ (residues 893–1,306), A-LC-H_N_ (residues 1–874, GenBank no. M30196), and B-LC-H_N_ (residues 1–860, GenBank no. AB232927) were synthesized by GenScript (New Brunswick, NJ, USA). The cDNA encoding X-H_N_ was generated in-house using the Gibson assembly method. X-LC, A-LC (residues 1–425) and B-LC (residues 1–439) were cloned into pET28 vectors with His6-tag on their N-termini. X-H_C_ and A-H_C_ (residues 875–1,297, GenBank No. AF488749) were cloned into pGEX4T to express as GST-tagged proteins. One extra glycine was introduced into the N terminus of X-H_C_ to increase the sortase ligation efficiency. X-LC-H_N_, A-LC-H_N_ and B-LC-H_N_ were cloned into pET22b vector, with the peptide sequence LPETGG fused to their C-termini, followed by a His6-tag, and purified as His6-tagged proteins. The cDNA encoding rat VAMP2 was generously provided by E. Chapman (Madison, WI, USA). VAMP2 (1–93) was cloned into pET28 vector with a His6-tag on the N terminus. VAMP2 (33–86) was cloned into pGEX4T vector and expressed as a GST-tagged protein. The cDNAs encoding mouse VAMP1, VAMP3, VAMP4, human VAMP5, rat VAMP7 and VAMP8 were generously provided by C. Hu (Louisville, KY, USA). Full-length VAMP1, 3, 7 and 8 were cloned into modified pcDNA3.1 vectors, with an HA tag fused to their C termini. Constructs expressing full-length rat Ykt6 and mouse Sec22b, both in pcDNA3.1 vector with a Myc tag fused to the N terminus of the protein, were generously provided by J. Hay (Missoula, MT, USA). The cytoplasmic domains of VAMP4 (1–115) and VAMP5 (1–70) were cloned between BamHI/XhoI sites in pGEX4T and expressed as GST-tagged proteins. We note that there are seven extra residues from the pGEX4T vector fused to the C-termini of VAMP4 and VAMP5 fragments. Ykt6 fragment (residues 1–192) was also cloned into pGEX4T and expressed as GST-tagged proteins. The construct encoding His6-tagged sortase (SrtA*) was generously provided by B. Pentelute (Boston, MA, USA)^43^.

### Bioinformatics

The Uniprot database was searched with Jackhmmer on the HMMER web server, using a BoNT/A1 sequence as the seed (Uniprot accession number A5HZZ9) until convergence. Returned sequences were aligned with Clustal Omega and a NeighborNet phylogenetic network estimated with SplitsTree.

### Protein purification

*E. coli* BL21 (DE3) was utilized for protein expression. In general, induction of expression was carried out with 0.1 mM IPTG at 22 °C overnight. Bacterial pellets were disrupted in lysis buffer (50 mM Tris pH 7.5, 150 mM NaCl) by sonication, and supernatants were collected after centrifugation at 20,000 *g* for 30 min at 4 °C. Protein purification was carried out using AKTA Prime FPLC system (GE), and purified proteins were further desalted with a PD-10 column (GE, 17-0851-01).

### Large-scale production and purification of BoNT/X_RY_

cDNA encoding BoNT/X_RY_ was assembled in-house from mutated X-LC (R360A/Y363F), X-H_N_ and X-H_C_. It was cloned into a pET22b vector, with the His6-tag on its C terminus. The corresponding plasmid was transformed into *E. coli* BL21 (DE3). Cultures for expression were first grown using a LEX Bioreactor (Epiphyte3, Ontario, Canada) at 37 °C in 1.5 l of medium until OD_600_ reached 0.8. The temperature was then reduced to 18 °C for induction of expression with 1 mM IPTG, and grown for 16–17 h. Bacteria were harvested, re-suspended in HEPES buffer (50 mM HEPES pH 7.2, 500 mM NaCl, 25 mM imidazole, 5% glycerol, 2 mM TCEP), and lysed with an Emulsiflex-C3 (Avestin, Mannheim, Germany) at 20,000 p.s.i. Lysates were ultra-centrifuged at 200,000 *g* for 45 min. Supernatant was loaded onto a 15 ml Ni-NTA agarose column and washed with wash buffer (50 mM HEPES pH7.2, 500 mM NaCl, 100 mM imidazole, 5% glycerol, 1 mM TCEP). Proteins were eluted with elution buffer (50 mM HEPES pH 7.2, 500 mM NaCl, 250 mM imidazole, 5% glycerol, 1 mM TCEP) and then dialyzed overnight in 50 mM HEPES, 500 mM NaCl, 5% glycerol, and 0.5 mM TCEP. Dialysate was concentrated using a Vivaspin concentrator before being loaded on a Superdex200-16/60 column pre-equilibrated in the same buffer used for dialysis. Elution peak corresponding to BoNT/X was collected and concentrated to ∼10 mg ml^−1^. Sample was aliquoted and flash-frozen in liquid nitrogen for storage at −80 °C.

### Cleavage of SNARE proteins in rat BDE

Rat brain was homogenized in 15 ml 320 mM sucrose buffer, followed by centrifugation at 5,000 r.p.m. for 2 min at 4 °C. Supernatants were collected and centrifuged at 11,000 r.p.m. for 12 min. The pellet was collected and solubilized for 30 min in 15 ml Tris-buffered saline (TBS: 20 mM Tris, 150 mM NaCl) plus 2% of Triton X-100 and a cocktail of protease inhibitors (Roche, CA). Samples were subsequently centrifuged at 17,000 r.p.m. for 20 min to remove insoluble materials. The final BDE concentration was ∼2 mg ml^−1^. BDE (60 μl) were incubated with X-LC (0.5 μM), A-LC (1 μM), or B-LC (1 μM), for 1 h at 37 °C, and then analysed by immunoblot using the enhanced chemiluminescence (ECL) method (Pierce). As controls, LCs were pre-incubated with 20 mM EDTA for 20 min at room temperature before adding to BDE. Full-blot scans are shown in Supplementary Fig. 9.

### Cleavage of recombinant VAMPs by X-LC

VAMP2 (1–93) was expressed and purified as a His6-tagged protein. VAMP2 (33–86), VAMP4 (1–115), VAMP5 (1–70) and Ykt6 (1–192) were expressed and purified as GST-tagged proteins. These proteins (0.3 mg ml^−1^) were incubated with 0.1 μM X-LC in TBS buffer at 37 °C. Samples were either analysed by SDS–PAGE gels and Coomassie Blue staining, or subjected to mass spectrometry analysis.

Cleavage of VAMPs in cell lysates: Full-length HA-tagged VAMP1, 3, 7, 8 and Myc-tagged Sec22b, and Ykt6 were transfected into 293T cells using PolyJet transfection reagents (SignaGen, MD) following the manufacturer’s instructions. Cell lysates were harvested 48 h later in RIPA buffer (50 mM Tris, 1% NP40, 150 mM NaCl, 0.5% sodium deoxycholate, 0.1% SDS, 400 μl per 10-cm dish) plus a protease inhibitor cocktail (Sigma-Aldrich). Cell lysates (250 μl) were incubated with X-LC (0.5 μM) for 1 h at 37 °C. Samples were then analysed by immunoblot.

### Identification of cleavage sites in VAMPs by LC–MS/MS

Samples were analysed at Taplin Biological Mass Spectrometry Core Facility at Harvard Medical School. For VAMP2, whole-protein samples were loaded onto a 100 μm internal diameter C18 reverse-phase HPLC column packed with 3 cm of beads off-line using a pressure cell. The column was re-attached to an Accela 600 Pump (Thermo Fisher Scientific). A rapid gradient of increasing acetonitrile was used to elute the protein/peptide from the HPLC column. As peptides eluted, they were subjected to electrospray ionization and then placed into an LTQ Orbitrap Velos Pro ion-trap mass spectrometer to acquire a high-resolution FTMS scan at 60,000 resolution, a second scan at low resolution in the ion trap, and a final scan to perform data-dependent MS/MS. The charge state envelopes were de-convoluted manually to obtain mono-isotopic masses when possible or average masses for the proteins. Peptide and protein identity were determined by matching protein databases with the acquired fragmentation pattern using the software program Sequest (Thermo Fisher Scientific). All databases include a reversed version of all the sequences, and the data were filtered to 1–2% peptide false-discovery rate.

For Ykt6, VAMP4 and VAMP5, samples were first separated on SDS–PAGE. Protein bands were excised and cut into ∼1 mm^3^ pieces. Gel pieces were incubated with 50 mM ammonium bicarbonate solution containing 12.5 ng μl^−1^ modified sequencing-grade chymotrypsin (Roche Diagnostics). Samples were digested overnight at room temperature. Peptides were then extracted and separated with reverse-phase HPLC. As peptides were eluted, they were subjected to electrospray ionization and transferred into an LTQ Orbitrap Velos Pro ion-trap mass spectrometer (Thermo Fisher Scientific). Eluted peptides were detected, isolated and fragmented to produce a tandem mass spectrum of specific fragment ions for each peptide.

### Identification of the protease cleavage site between LC and H_N_

His6-tagged recombinant X-LC-H_N_ fragment (residues 1–891) was purified in *E. coli* and subjected to limited proteolysis by endoproteinase Lys-C (Sigma P2289, 100:1 (toxin:Lys-C) molar ratio, 25 min at room temperature). The cleavage site was determined by TMT labelling and tandem mass spectrometry. Briefly, intact X-LC-H_N_ samples were labelled with the light TMT, and equal amounts of X-LC-H_N_ samples treated with Lys-C were labelled with the heavy TMT. Both samples were then digested with chymotrypsin, combined and subjected to quantitative mass spectrometry analysis.

### Cysteine alkylation by NEM

Lys-C-activated X-LC-H_N_ fragment was diluted into sodium phosphate buffer (10 mM, pH 6.5) at a final concentration of 0.3 mg ml^−1^, with or without NEM at indicated concentrations (20, 10 and 5 mM) and incubated for 10 min at room temperature. NEM was freshly prepared in sodium phosphate buffer. Samples were mixed with 3 × neutral loading dye (200 mM Tris pH 6.8, 30% glycerol, 6% Lithium Dodecyl sulfate, 10 mM NEM, and 0.06% BPB) at room temperature for 10 min, heated for 10 min at 55 °C, and then analysed by SDS–PAGE and Coomassie Blue staining.

### Neuron culture and immunoblot analysis

Primary rat cortical neurons were prepared from E18-19 embryos using a papain dissociation kit (Worthington Biochemical) following the manufacturer’s instruction^57^. Neurons were exposed to either BoNT/X fragments or sortase ligation mixtures in culture medium for 12 h. Cells were then lysed with RIPA buffer plus a protease inhibitor cocktail (Sigma-Aldrich). Lysates were centrifuged for 10 min at maximum speed using a microcentrifuge at 4 °C. Supernatants were subjected to SDS–PAGE and immunoblot analysis.

### Dot blot

BoNTs (0.2 μg in 1 μl) were spotted onto nitrocellulose membranes and dried (10 min at RT). The membranes were blocked with 5% milk in TBST (TBS plus 0.05% Tween20) for 30 min and then incubated with appropriate antisera (1:500 dilution) for 30 min. The membranes were then washed three times with TBST and incubated with HRP (horseradish peroxidase)-conjugated secondary antibodies for 30 min, washed three more times with TBST, and analysed by the ECL method. The BoNT/X sample was composed of X-LC-H_N_ and GST-X-H_C_ at 1:1 molar ratio.

### Sortase-mediated ligation

GST-X-H_C_ or GST-A-H_C_ was cleaved overnight at 4 °C by thrombin before being added into the ligation reaction mixture. Ligation reaction was set up in 50 μl TBS buffer with X-LC-H_N_ (8 μM), X-H_C_ (4 μM) or A-H_C_ (25 μM), Ca^2+^ (10 mM) and sortase (10 μM), for 40 min at room temperature.

### DAS assay

All procedures were conducted in accordance with the guidelines approved by the Institute Animal Care and Use Committee (IACUC) at Boston Children’s Hospital (#3030). Briefly, X-FL generated by sortase-mediated ligation was first activated with limited proteolysis using trypsin (60:1 (toxin:trypsin) molar ratio, 30 min at room temperature). We chose trypsin instead of Lys-C here, as it allows us to stop proteolysis by adding trypsin inhibitors (Soybean trypsin inhibitor, 1:10 ratio (trypsin:trypsin inhibitor). Mice (CD-1 strain, male, purchased from Charles River, 5–6 weeks old, 21–25 g, *n*=4) were anesthetized with isoflurane (3–4%) and injected with X-FL (0.5 μg) using a 30-gauge needle attached to a sterile Hamilton syringe, into the gastrocnemius muscles of the right hind limb. Muscle paralysis and the spread of hind paw in response to a startle stimulus were observed 12 h after injection as previously described^44^.

### Biosafety and biosecurity

All procedures were approved by the Institute of Biosafety Committees at Boston Children’s Hospital and at Stockholm University. To ensure biosafety and biosecurity, no active full-length toxin gene was produced in any form. The amount of sortase linking reaction is strictly controlled to ensure that only a minimal amount of ligated toxins was produced, which was immediately utilized for functional studies. The loss of toxicity of BoNT/X_RY_ and reduced toxicity of sortase-linked X-FL were confirmed using the mouse lethality assay. Inactive BoNT/X_RY_ was prepared and is available for developing neutralizing antibodies.

### Data availability

The data and materials that support the findings of this study are available from the corresponding authors upon request.

## Additional information

**How to cite this article:** Zhang, S. *et al*. Identification and characterization of a novel botulinum neurotoxin. *Nat. Commun.* **8**, 14130 doi: 10.1038/ncomms14130 (2017).

**Publisher’s note:** Springer Nature remains neutral with regard to jurisdictional claims in published maps and institutional affiliations.

## Supplementary Material

Supplementary Information Supplementary Figures

Supplementary Data 1 Peptide fragments of X-LC-HN under limited proteolysis analyzed by TMT labeling and quantitative mass spectrometry. His6-tagged recombinant X-LC-HN was labeled with the light TMT. Equal amounts of X-LC-HN samples were exposed to Lys-C and then labeled with the heavy TMT. Both samples were then digested with chymotrypsin, combined, and subjected to quantitative mass spectrometry. A list of identified peptides is shown in the associated Excel file. The light TMT: heavy TMT ratios are within 2-fold of each other for all except five peptides (marked in red), all of which start with N439, indicating that N439 is a new N-terminus generated by Lys-C cutting.

## References

[b1] Arnon S. S. . Botulinum toxin as a biological weapon: medical and public health management. JAMA 285, 1059–1070 (2001). 11209178 10.1001/jama.285.8.1059

[b2] Johnson E. A. Clostridial toxins as therapeutic agents: benefits of nature’s most toxic proteins. Annu. Rev. Microbiol. 53, 551–575 (1999). 10547701 10.1146/annurev.micro.53.1.551

[b3] Montecucco C. & Molgo J. Botulinal neurotoxins: revival of an old killer. Curr. Opin. Pharmacol. 5, 274–279 (2005). 15907915 10.1016/j.coph.2004.12.006

[b4] Schiavo G., Matteoli M. & Montecucco C. Neurotoxins affecting neuroexocytosis. Physiol. Rev. 80, 717–766 (2000). 10747206 10.1152/physrev.2000.80.2.717

[b5] Montal M. Botulinum neurotoxin: a marvel of protein design. Annu. Rev. Biochem. 79, 591–617 (2010). 20233039 10.1146/annurev.biochem.051908.125345

[b6] Rossetto O., Pirazzini M. & Montecucco C. Botulinum neurotoxins: genetic, structural and mechanistic insights. Nat. Rev. Microbiol. 12, 535–549 (2014). 24975322 10.1038/nrmicro3295

[b7] Jahn R. & Scheller R. H. SNAREs--engines for membrane fusion. Nat. Rev. Mol. Cell Biol. 7, 631–643 (2006). 16912714 10.1038/nrm2002

[b8] Sudhof T. C. & Rothman J. E. Membrane fusion: grappling with SNARE and SM proteins. Science 323, 474–477 (2009). 19164740 10.1126/science.1161748 PMC3736821

[b9] Burke G. S. Notes on Bacillus botulinus. J. Bacteriol. 4, 555–570 (1919). 16558852 10.1128/jb.4.5.555-570.1.1919 PMC378820

[b10] Gimenez D. F. & Ciccarelli A. S. Another type of *Clostridium botulinum*. Zentralbl Bakteriol. Orig. 215, 221–224 (1970). 4922309

[b11] Smith T. J. . Sequence variation within botulinum neurotoxin serotypes impacts antibody binding and neutralization. Infect. Immun. 73, 5450–5457 (2005). 16113261 10.1128/IAI.73.9.5450-5457.2005 PMC1231122

[b12] Hill K. K. . Genetic diversity among Botulinum Neurotoxin-producing clostridial strains. J. Bacteriol. 189, 818–832 (2007). 17114256 10.1128/JB.01180-06 PMC1797315

[b13] Montecucco C. & Rasotto M. B. On botulinum neurotoxin variability. MBio. 6, e02131–e02134 (2015). 25564463 10.1128/mBio.02131-14 PMC4313909

[b14] Dover N., Barash J. R., Hill K. K., Xie G. & Arnon S. S. Molecular characterization of a novel botulinum neurotoxin type H gene. J. Infect. Dis. 209, 192–202 (2014). 24106295 10.1093/infdis/jit450

[b15] Barash J. R. & Arnon S. S. A novel strain of *Clostridium botulinum* that produces type B and type H botulinum toxins. J. Infect. Dis. 209, 183–191 (2014). 24106296 10.1093/infdis/jit449

[b16] Maslanka S. E. . A novel botulinum neurotoxin, previously reported as serotype H, has a hybrid-like structure with regions of similarity to the structures of serotypes A and F and is neutralized with serotype A antitoxin. J. Infect. Dis. 213, 379–385 (2015). 26068781 10.1093/infdis/jiv327 PMC4704661

[b17] Kalb S. R. . Functional characterization of botulinum neurotoxin serotype H as a hybrid of known serotypes F and A (BoNT F/A). Anal. Chem. 87, 3911–3917 (2015). 25731972 10.1021/ac504716v PMC4522910

[b18] Zhou Y., Sugiyama H., Nakano H. & Johnson E. A. The genes for the *Clostridium botulinum* type G toxin complex are on a plasmid. Infect. Immun. 63, 2087–2091 (1995). 7729925 10.1128/iai.63.5.2087-2091.1995 PMC173270

[b19] Marshall K. M., Bradshaw M., Pellett S. & Johnson E. A. Plasmid encoded neurotoxin genes in *Clostridium botulinum* serotype A subtypes. Biochem. Biophys. Res. Commun. 361, 49–54 (2007). 17658467 10.1016/j.bbrc.2007.06.166 PMC2346372

[b20] Jacobson M. J., Lin G., Raphael B., Andreadis J. & Johnson E. A. Analysis of neurotoxin cluster genes in *Clostridium botulinum* strains producing botulinum neurotoxin serotype A subtypes. Appl. Environ. Microbiol. 74, 2778–2786 (2008). 18326685 10.1128/AEM.02828-07 PMC2394882

[b21] Eklund M. W., Poysky F. T., Reed S. M. & Smith C. A. Bacteriophage and the toxigenicity of *Clostridium botulinum* type C. Science 172, 480–482 (1971). 4927679 10.1126/science.172.3982.480

[b22] Smith T. J. . Analysis of the neurotoxin complex genes in *Clostridium botulinum* A1-A4 and B1 strains: BoNT/A3, /Ba4 and /B1 clusters are located within plasmids. PLoS ONE 2, e1271 (2007). 18060065 10.1371/journal.pone.0001271 PMC2092393

[b23] Hill K. K., Xie G., Foley B. T. & Smith T. J. Genetic diversity within the botulinum neurotoxin-producing bacteria and their neurotoxins. Toxicon 107, 2–8 (2015). 26368006 10.1016/j.toxicon.2015.09.011

[b24] Luquez C., Raphael B. H. & Maslanka S. E. Neurotoxin gene clusters in *Clostridium botulinum* type Ab strains. Appl. Environ. Microbiol. 75, 6094–6101 (2009). 19684172 10.1128/AEM.01009-09 PMC2753052

[b25] Dover N. . *Clostridium botulinum* strain Af84 contains three neurotoxin gene clusters: bont/A2, bont/F4 and bont/F5. PLoS ONE 8, e61205 (2013). 23637798 10.1371/journal.pone.0061205 PMC3625220

[b26] Franciosa G., Ferreira J. L. & Hatheway C. L. Detection of type A, B, and E botulism neurotoxin genes in *Clostridium botulinum* and other Clostridium species by PCR: evidence of unexpressed type B toxin genes in type A toxigenic organisms. J. Clin. Microbiol. 32, 1911–1917 (1994). 7989542 10.1128/jcm.32.8.1911-1917.1994 PMC263902

[b27] Hutson R. A. . Genetic characterization of *Clostridium botulinum* type A containing silent type B neurotoxin gene sequences. J. Biol. Chem. 271, 10786–10792 (1996). 8631890 10.1074/jbc.271.18.10786

[b28] Dover N., Barash J. R. & Arnon S. S. Identical novel A5(B3') botulinum neurotoxin gene arrangements isolated from widely disparate geographical and patient sources suggest their independent origins. J. Clin. Microbiol. 48, 1989 (2010). 20444983 10.1128/JCM.00468-10 PMC2863925

[b29] Dabritz H. A. . Molecular epidemiology of infant botulism in California and elsewhere, 1976–2010. J. Infect. Dis. 210, 1711–1722 (2014). 24924163 10.1093/infdis/jiu331

[b30] Kakinuma H., Maruyama H., Takahashi H., Yamakawa K. & Nakamura S. The first case of type B infant botulism in Japan. Acta Paediatr. Jpn 38, 541–543 (1996). 8942019 10.1111/j.1442-200x.1996.tb03542.x

[b31] Kozaki S. . Characterization of *Clostridium botulinum* type B neurotoxin associated with infant botulism in japan. Infect. Immun. 66, 4811–4816 (1998). 9746583 10.1128/iai.66.10.4811-4816.1998 PMC108594

[b32] Ihara H. . Sequence of the gene for *Clostridium botulinum* type B neurotoxin associated with infant botulism, expression of the C-terminal half of heavy chain and its binding activity. Biochim. Biophys. Acta 1625, 19–26 (2003). 12527421 10.1016/s0167-4781(02)00537-7

[b33] Schiavo G. . Tetanus and botulinum-B neurotoxins block neurotransmitter release by proteolytic cleavage of synaptobrevin. Nature 359, 832–835 (1992). 1331807 10.1038/359832a0

[b34] Rummel A., Mahrhold S., Bigalke H. & Binz T. The HCC-domain of botulinum neurotoxins A and B exhibits a singular ganglioside binding site displaying serotype specific carbohydrate interaction. Mol. Microbiol. 51, 631–643 (2004). 14731268 10.1046/j.1365-2958.2003.03872.x

[b35] Gu S. . Botulinum neurotoxin is shielded by NTNHA in an interlocked complex. Science 335, 977–981 (2012). 22363010 10.1126/science.1214270 PMC3545708

[b36] Lee K. . Molecular basis for disruption of E-cadherin adhesion by botulinum neurotoxin A complex. Science 344, 1405–1410 (2014). 24948737 10.1126/science.1253823 PMC4164303

[b37] Lee K. . Structure of a bimodular botulinum neurotoxin complex provides insights into its oral toxicity. PLoS Pathog. 9, e1003690 (2013). 24130488 10.1371/journal.ppat.1003690 PMC3795040

[b38] Sugawara Y. . Botulinum hemagglutinin disrupts the intercellular epithelial barrier by directly binding E-cadherin. J. Cell Biol. 189, 691–700 (2010). 20457762 10.1083/jcb.200910119 PMC2872904

[b39] Rossetto O. . SNARE motif and neurotoxins. Nature 372, 415–416 (1994). 7984234 10.1038/372415a0

[b40] Yamamoto H. . Specificity of botulinum protease for human VAMP family proteins. Microbiol. Immunol. 56, 245–253 (2012). 22289120 10.1111/j.1348-0421.2012.00434.x

[b41] Chaddock J. A. . Expression and purification of catalytically active, non-toxic endopeptidase derivatives of *Clostridium botulinum* toxin type A. Protein Expr. Purif. 25, 219–228 (2002). 12135553 10.1016/s1046-5928(02)00002-5

[b42] Popp M. W., Antos J. M., Grotenbreg G. M., Spooner E. & Ploegh H. L. Sortagging: a versatile method for protein labeling. Nat. Chem. Biol. 3, 707–708 (2007). 17891153 10.1038/nchembio.2007.31

[b43] McCluskey A. J. & Collier R. J. Receptor-directed chimeric toxins created by sortase-mediated protein fusion. Mol. Cancer Ther. 12, 2273–2281 (2013). 23945077 10.1158/1535-7163.MCT-13-0358 PMC3795991

[b44] Aoki K. R. A comparison of the safety margins of botulinum neurotoxin serotypes A, B, and F in mice. Toxicon 39, 1815–1820 (2001). 11600142 10.1016/s0041-0101(01)00101-5

[b45] Binz T., Bade S., Rummel A., Kollewe A. & Alves J. Arg(362) and Tyr(365) of the botulinum neurotoxin type a light chain are involved in transition state stabilization. Biochemistry 41, 1717–1723 (2002). 11827515 10.1021/bi0157969

[b46] Pier C. L. . Recombinant holotoxoid vaccine against botulism. Infect. Immun. 76, 437–442 (2008). 17967862 10.1128/IAI.00843-07 PMC2223665

[b47] Steegmaier M., Klumperman J., Foletti D. L., Yoo J. S. & Scheller R. H. Vesicle-associated membrane protein 4 is implicated in trans-Golgi network vesicle trafficking. Mol. Biol. Cell 10, 1957–1972 (1999). 10359608 10.1091/mbc.10.6.1957 PMC25394

[b48] Brandhorst D. . Homotypic fusion of early endosomes: SNAREs do not determine fusion specificity. Proc. Natl Acad. Sci. USA 103, 2701–2706 (2006). 16469845 10.1073/pnas.0511138103 PMC1413832

[b49] Daste F., Galli T. & Tareste D. Structure and function of longin SNAREs. J. Cell Sci. 128, 4263–4272 (2015). 26567219 10.1242/jcs.178574

[b50] Raingo J. . VAMP4 directs synaptic vesicles to a pool that selectively maintains asynchronous neurotransmission. Nat. Neurosci. 15, 738–745 (2012). 22406549 10.1038/nn.3067 PMC3337975

[b51] Cocucci E., Racchetti G., Rupnik M. & Meldolesi J. The regulated exocytosis of enlargeosomes is mediated by a SNARE machinery that includes VAMP4. J. Cell Sci. 121, 2983–2991 (2008). 18713833 10.1242/jcs.032029

[b52] Nicholson-Fish J. C., Kokotos A. C., Gillingwater T. H., Smillie K. J. & Cousin M. A. VAMP4 is an essential cargo molecule for activity-dependent bulk endocytosis. Neuron 88, 973–984 (2015). 26607000 10.1016/j.neuron.2015.10.043 PMC4678114

[b53] Cooper A. A. . Alpha-synuclein blocks ER-Golgi traffic and Rab1 rescues neuron loss in Parkinson’s models. Science 313, 324–328 (2006). 16794039 10.1126/science.1129462 PMC1983366

[b54] Thayanidhi N. . Alpha-synuclein delays endoplasmic reticulum (ER)-to-Golgi transport in mammalian cells by antagonizing ER/Golgi SNAREs. Mol. Biol. Cell 21, 1850–1863 (2010). 20392839 10.1091/mbc.E09-09-0801 PMC2877643

[b55] Zeng Q. . A novel synaptobrevin/VAMP homologous protein (VAMP5) is increased during *in vitro* myogenesis and present in the plasma membrane. Mol. Biol. Cell 9, 2423–2437 (1998). 9725904 10.1091/mbc.9.9.2423 PMC25509

[b56] Krzewski K., Gil-Krzewska A., Watts J., Stern J. N. & Strominger J. L. VAMP4- and VAMP7-expressing vesicles are both required for cytotoxic granule exocytosis in NK cells. Eur. J. Immunol. 41, 3323–3329 (2011). 21805468 10.1002/eji.201141582 PMC3438144

[b57] Peng L., Tepp W. H., Johnson E. A. & Dong M. Botulinum neurotoxin D uses synaptic vesicle protein SV2 and gangliosides as receptors. PLoS Pathog. 7, e1002008 (2011). 21483489 10.1371/journal.ppat.1002008 PMC3068998

