# Supplementary material for: Identification and characterization of a novel botulinum neurotoxin
Source: Nat Commun. 2017 Aug 3;8:14130. doi: 10.1038/ncomms14130 (PMC5543303; doi:10.1038/ncomms14130)

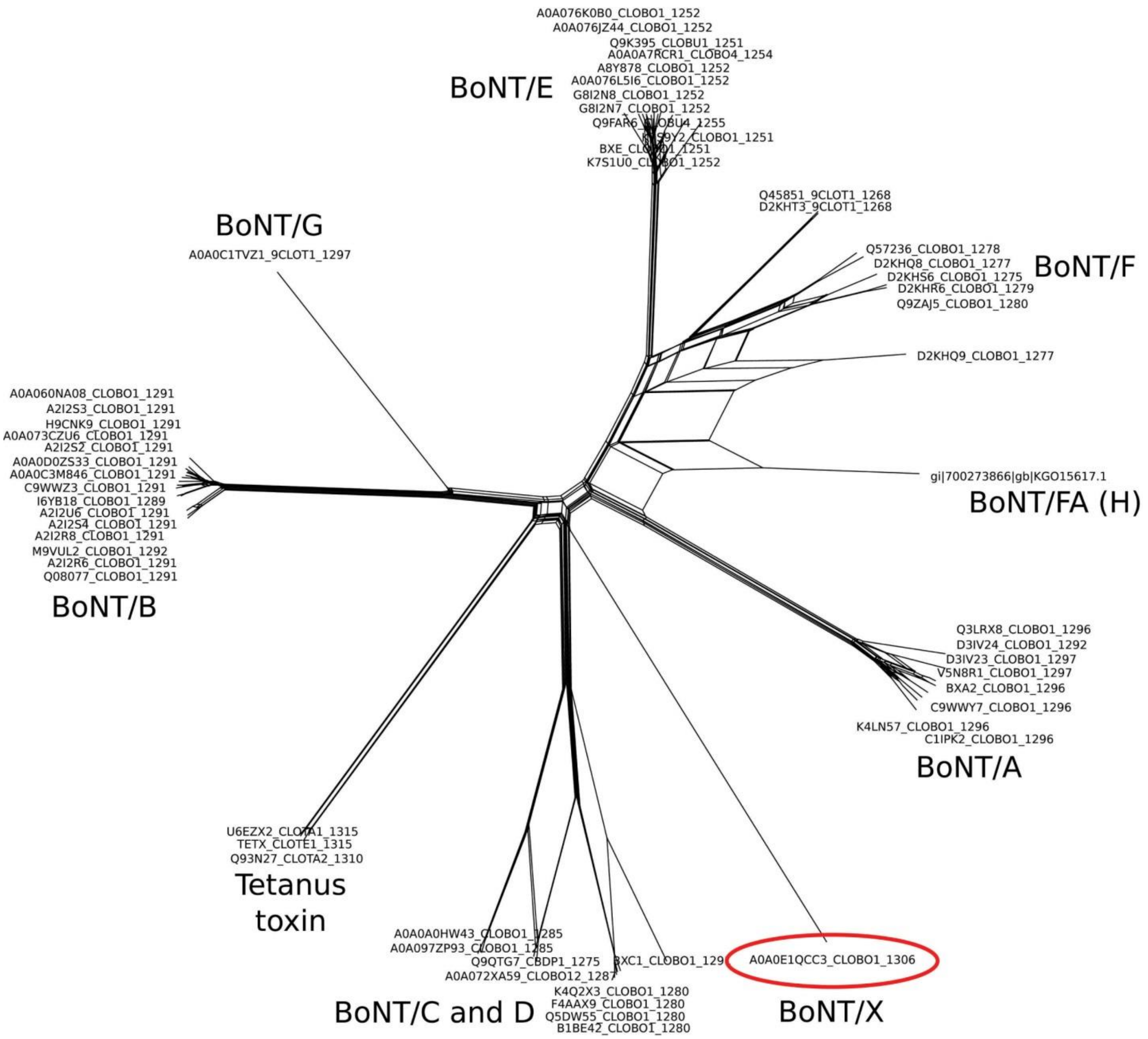

Supplementary Figure 1 | Phylogenetic split networks of clostridial neurotoxins.

The Uniprot database was searched with Jackhmmer using BoNT/A1 sequence as the seed until convergence. Returned sequences were aligned with Clustal Omega and a NeighborNet phylogenetic network estimated with SplitsTree. BoNT/X is circled in red. In contrast to phylogenetic trees, phylogenetic split networks visualize some of the conflict in the distance matrix, in the form of splits of the data. Each set of parallel edges connects a split; the lengths of the edges are proportional to the amount of support for a split. The access number for each identified toxin gene is noted.

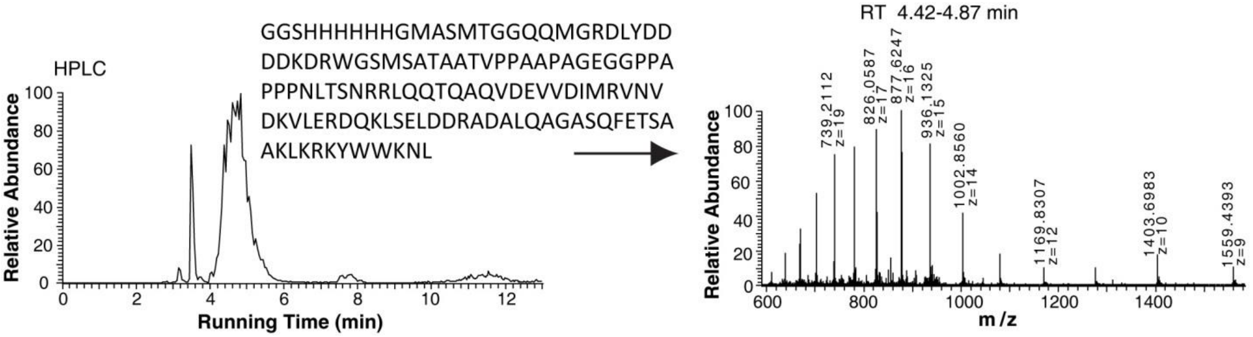

Supplementary Figure 2 | Mass spectrometry analysis of intact VAMP2 (1-93).

His6-tagged VAMP2 (1-93) was analyzed by LC-MS/MS mass spectrometry. The HPLC profile is presented in the left panel, together with the protein sequence. The mass spectrometry data for full-length VAMP2 (1-93) is shown in the right panel, with m/z values marked for each signal. RT: running time.

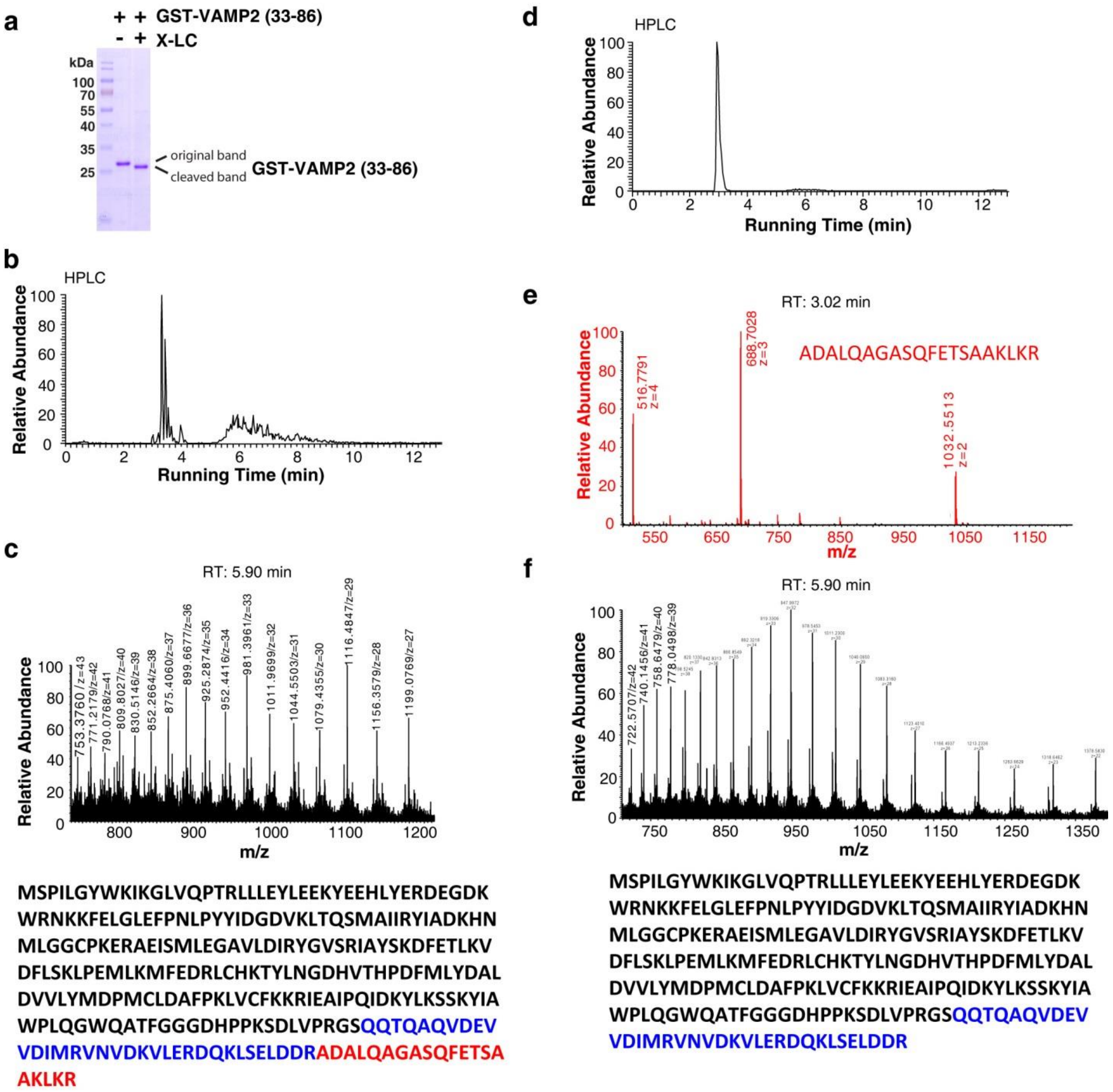

Supplementary Figure 3 | Identification of the cleavage site of BoNT/X on VAMP2 (33-86).

(**a**) GST-tagged VAMP2 (33-86) was incubated with or without X-LC. Samples were analyzed by SDS-PAGE and Coomassie Blue staining. (**b,c**) Intact GST-tagged VAMP2 (33-86) was analyzed by LC-MS/MS mass spectrometry. The blue color marks VAMP2 (33-66) and the red color marks VAMP2 (67-86). (**d-f)** GST-tagged VAMP2 (33-86) was incubated with X-LC. Samples were then analyzed by LC-MS/MS mass spectrometry. The HPLC profile is shown in (**d**), mass spectrometry data for the C-terminal fragment generated by X-LC in (**e**), and mass spectrometry data for the N-terminal fragment in (**f**).

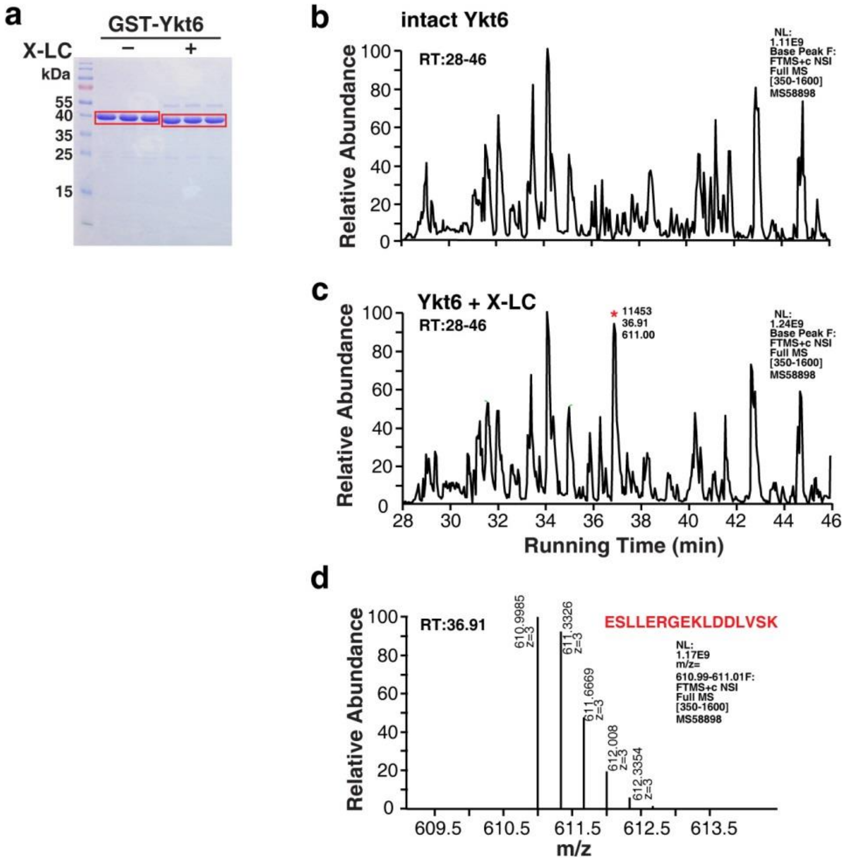

Supplementary Figure 4 | Identification of the cleavage site of BoNT/X on Ykt6.

(**a-d**) GST-tagged Ykt6 (residues 1-192, 10 µg), with or without pre-incubation with X-LC, were separated on SDS-PAGE (**a**). The protein bands were excised and digested by chymotrypsin. Digested peptides were desalted and analyzed by HPLC coupled with mass spectrometry. The HPLC profiles of GST-Ykt6 without pre-treatment with X-LC are shown in (**b**), and the sample pretreated with X-LC is shown in (**c**). One peptide (denoted with a red asterisk) was identified as ~100-fold more abundant in the samples pre-treated with X-LC compared to the samples that were not exposed to X-LC. This peptide was eluted at 37 min RT, with m/z = 611 as shown in (**d**), which can fit only the peptide sequence ESLLERGEKLDDLVSK in Ykt6, indicating that this is the peptide located at the N-terminal side of the cleavage site. Therefore the cleavage site is K173-S174 on Ykt6.

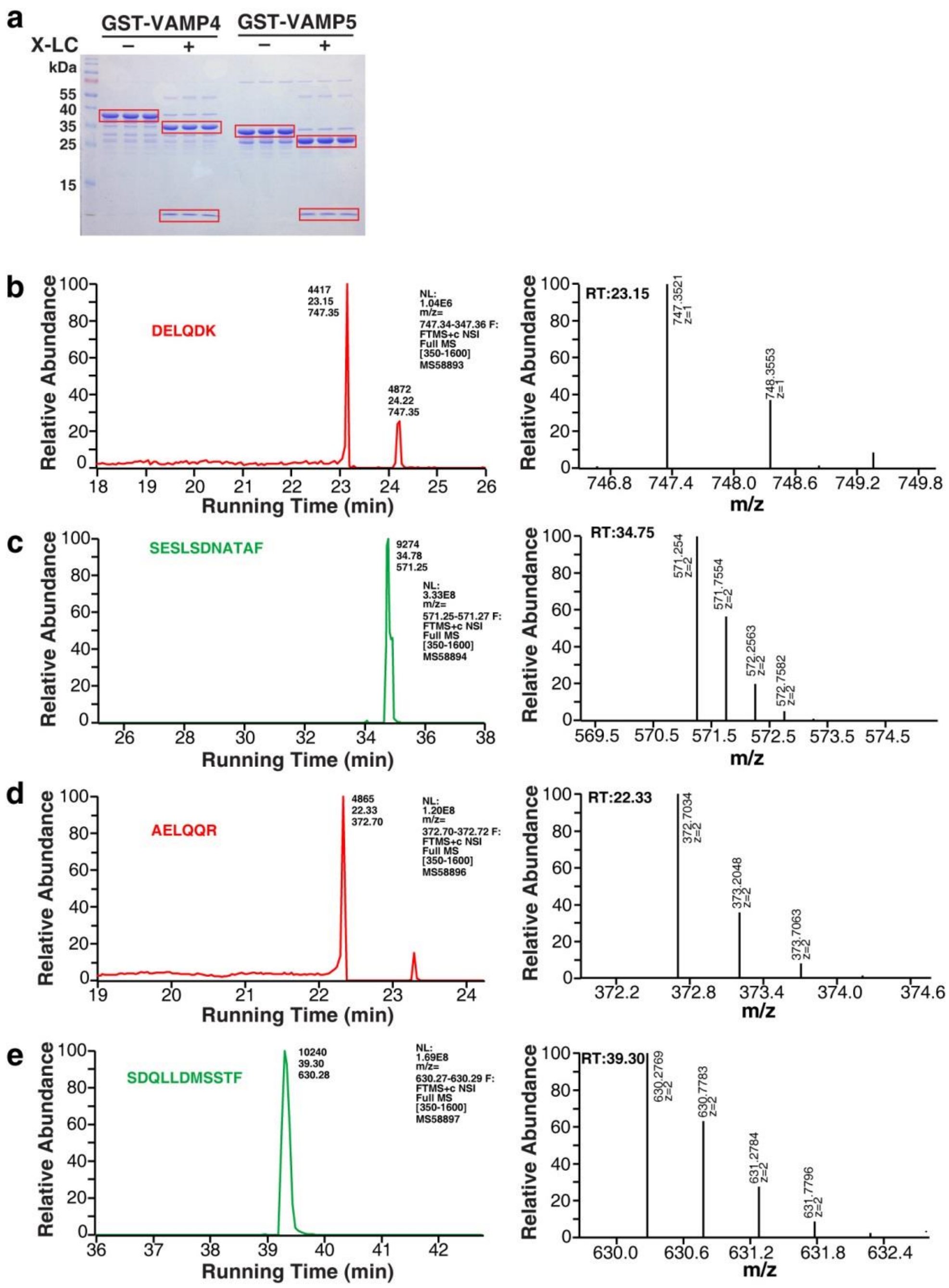

Supplementary Figure 5 | Identification of the cleavage sites of BoNT/X on VAMP4 and VAMP5.

(**a-e**) Experiments were carried out as described in Supplementary Fig. 4, except that VAMP4 and VAMP5 were analyzed here. The peptides that mark the N-terminal site of the cleavage site in VAMP4 (**b**), the C-terminal site of the cleavage site in VAMP4 (**c**), the N-terminal site of the cleavage site in VAMP5 (**d**), and the C-terminal site of the cleavage site in VAMP5 (**e**) are shown. The cleavage sites are determined to be K87-S88 in VAMP4 and R40-S41 in VAMP5.

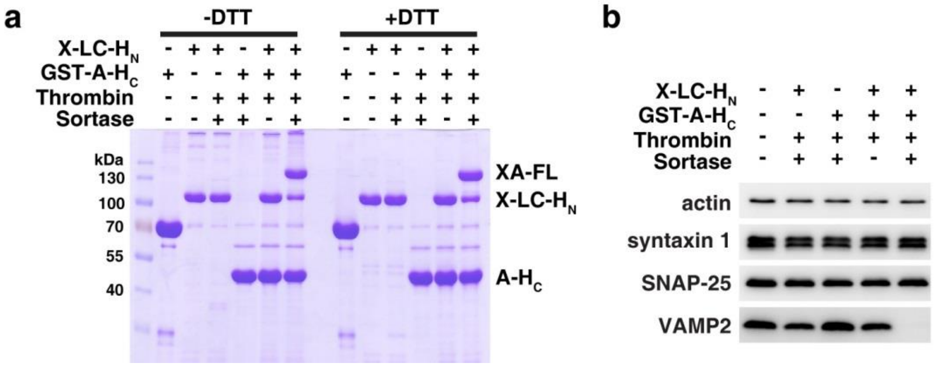

Supplementary Figure 6 | XA chimeric toxin is active on neurons.

(**a**) X-LC-H_N was ligated with A-H_C by sortase-mediated linking as described in Fig. 4a. The sortase ligation mixture and indicated control mixtures were analyzed by SDS-PAGE and Coomassie Blue staining. (**b**) Rat cortical neurons were exposed to indicated control mixtures or sortase-ligated XA mixture (5 µl) for 12 h in culture medium. Cell lysates were analyzed by immunoblot. X-LC-H_N alone cleaved some VAMP2 due to its high concentration in the reaction mixture. Ligated XA cleaved VAMP2 in neurons. One of two independent experiments is shown.

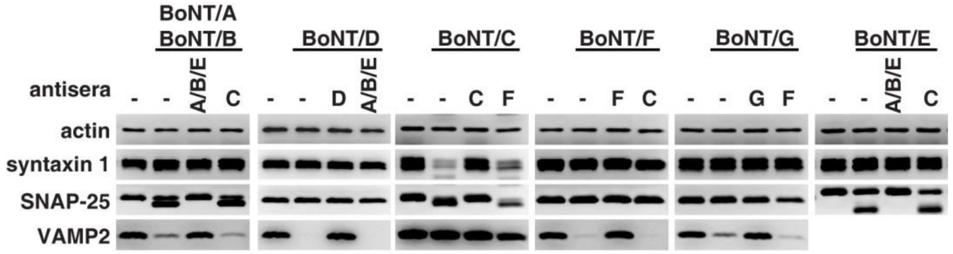

Supplementary Figure 7 | Antisera raised against the seven serotypes of BoNTs neutralized their target BoNTs on neurons.

Cultured rat cortical neurons were exposed to BoNTs, with or without pre-incubation with indicated antisera. Cell lysates were harvested 12 h later and subjected to immunoblot analysis. All antisera specifically neutralized their target BoNTs, without affecting the activity of a different serotype of BoNTs, thus validating their specificity and potency. The concentrations of BoNTs were: BoNT/A (50 pM), BoNT/B (2 nM), BoNT/C (1.5 nM), BoNT/D (100 pM), BoNT/E (0.5 nM), BoNT/F (0.5 nM), and BoNT/G (5 nM). The antiserum against BoNT/A/B/E was used at 20 µl per ml of culture medium (1:50 dilution). Other antisera were used at 1:100 dilution. BoNTs were pre-incubated with indicated antisera for 30 mins at 37 °C prior to adding to culture medium.

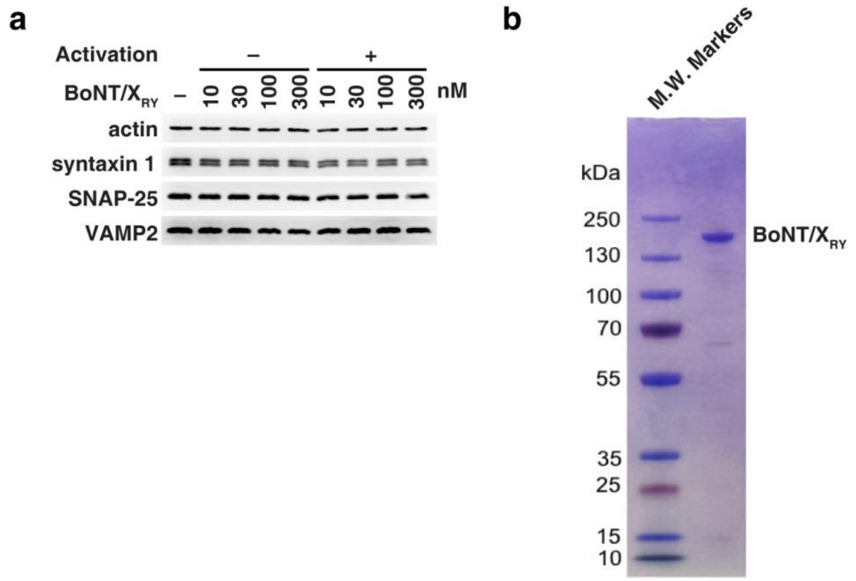

Supplementary Figure 8 | Production of an atoxic mutant BoNT/X$_{RY}$.

(**a**) Cultured rat cortical neurons were exposed to either single-chain or trypsin-activated di-chain BoNT/X$_{RY}$ at indicated concentrations. Cell lysates were analyzed by immunoblot. VAMP2 was not cleaved, indicating that BoNT/X$_{RY}$ is not active on neurons. Furthermore, intraperitoneal injection of mice with 30 µg activated BoNT/X$_{RY}$ did not cause any adverse effects (n = 5), demonstrating that it is not active. One of two independent experiments is shown. (**b**) Final sample of highly purified BoNT/X$_{RY}$ (in the presence of reducing agent) was analyzed by SDS-PAGE (4-12% BisTris).

Supplementary Figure 9 (full blots)

Figure 2a

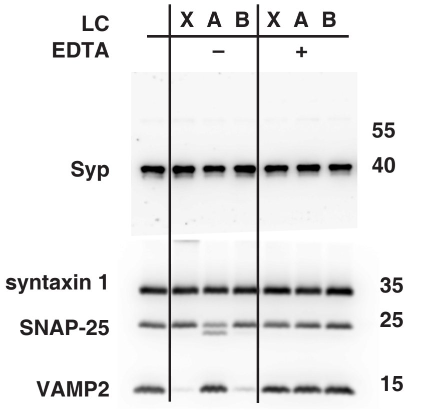

Figure 2g

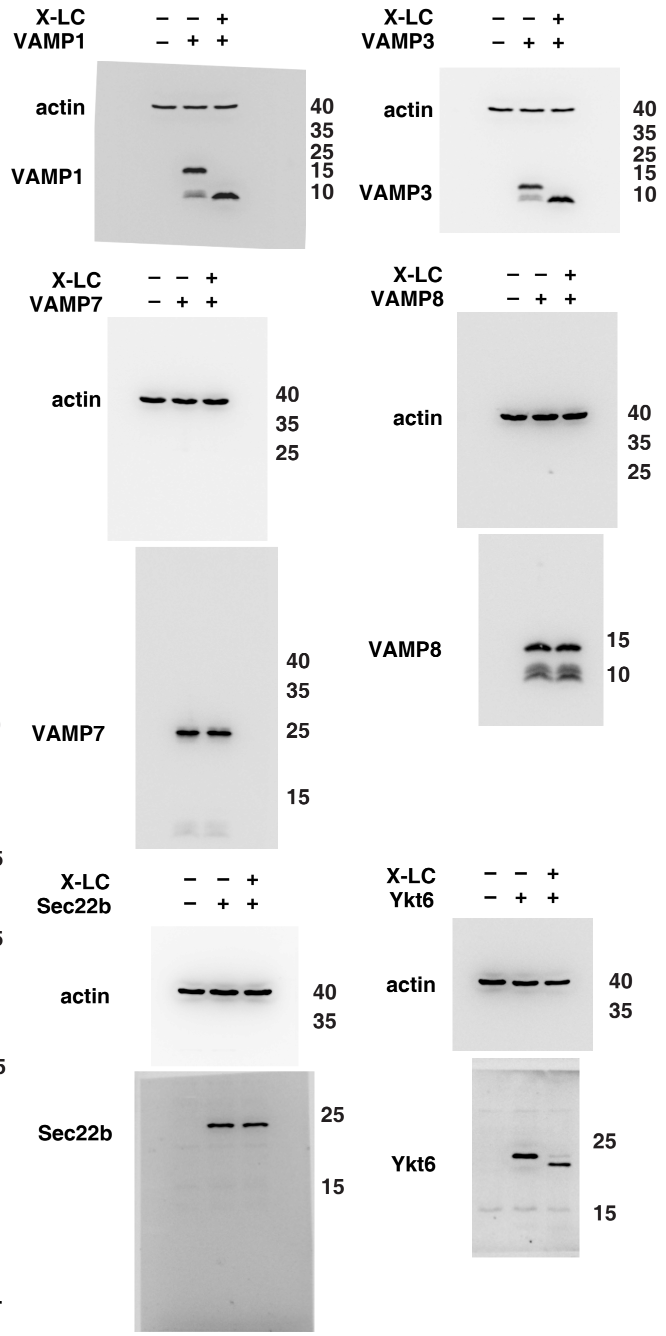

Figure 2j

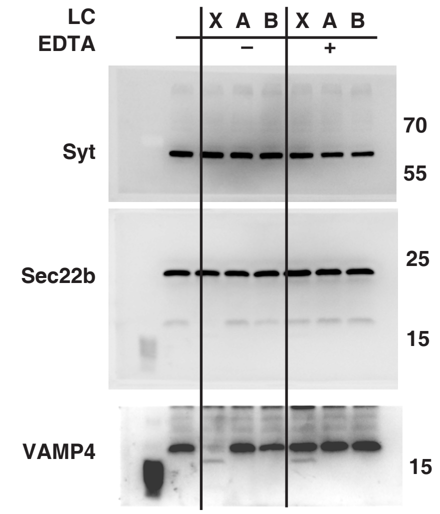

Figure 3c

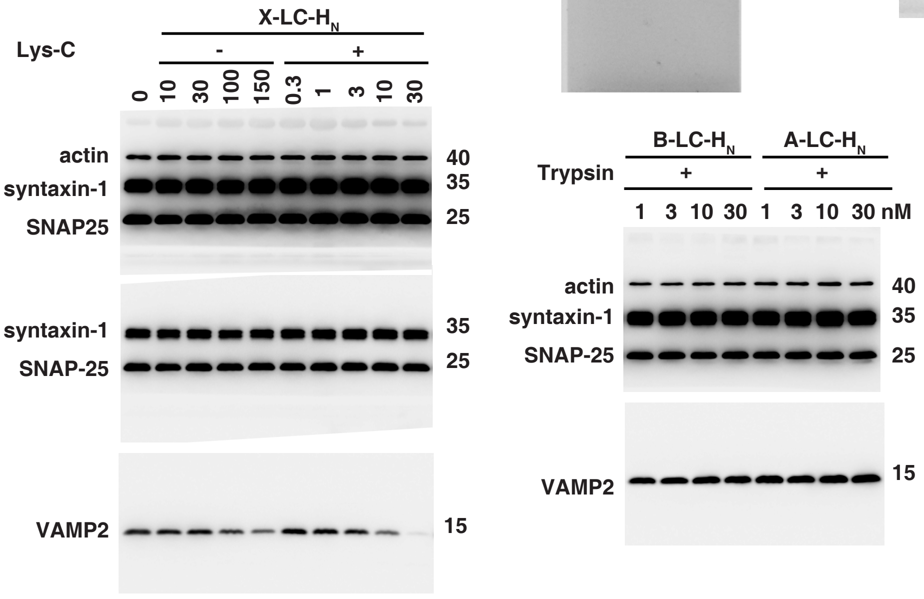

Figure 3f

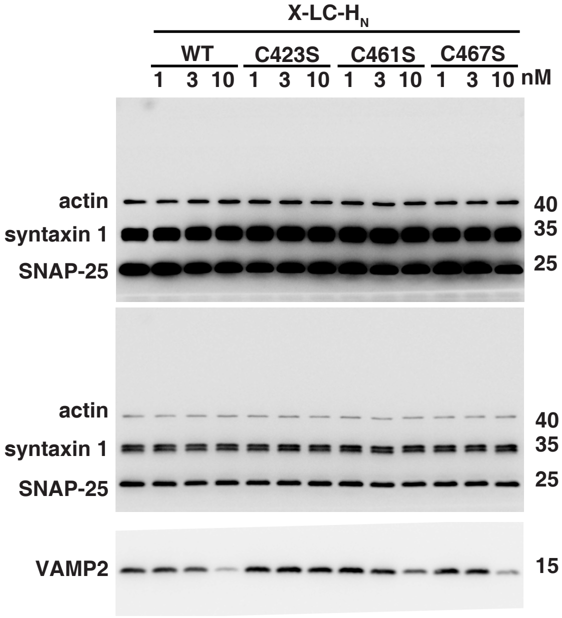

Figure 4c

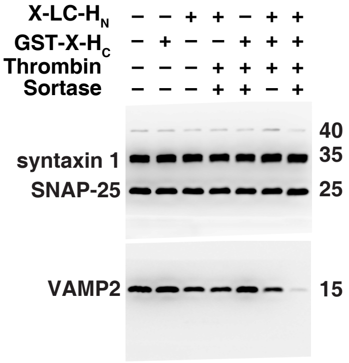

Figure 4e

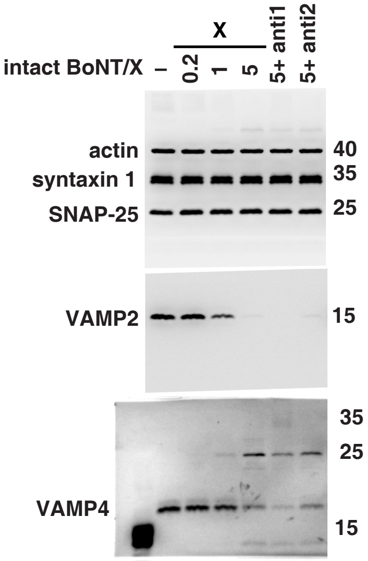

Figure 4d

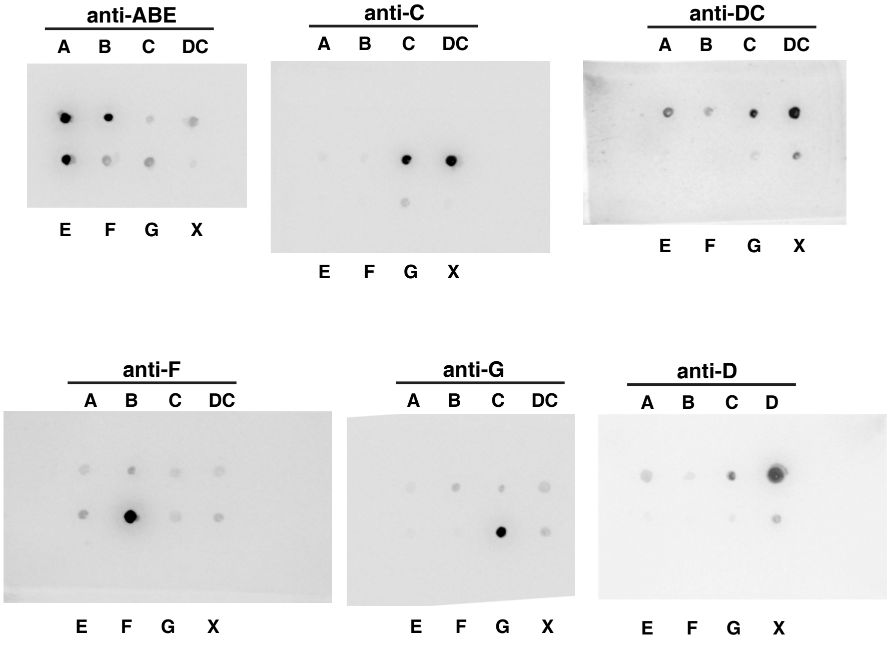

Supplementary figure 6

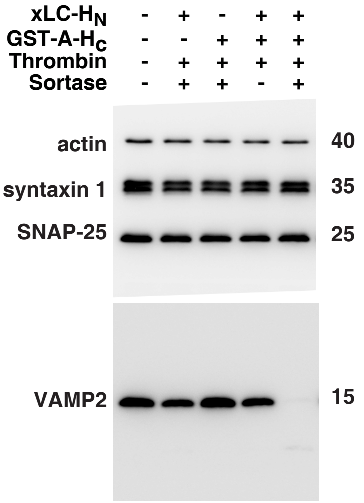

Supplementary figure 8

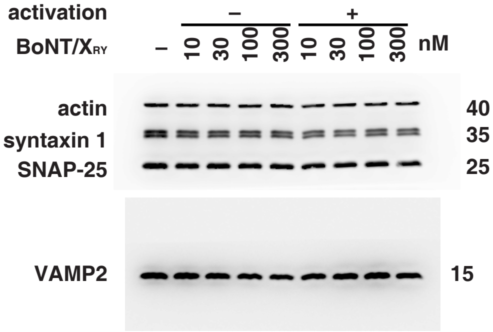

Supplementary figure 7

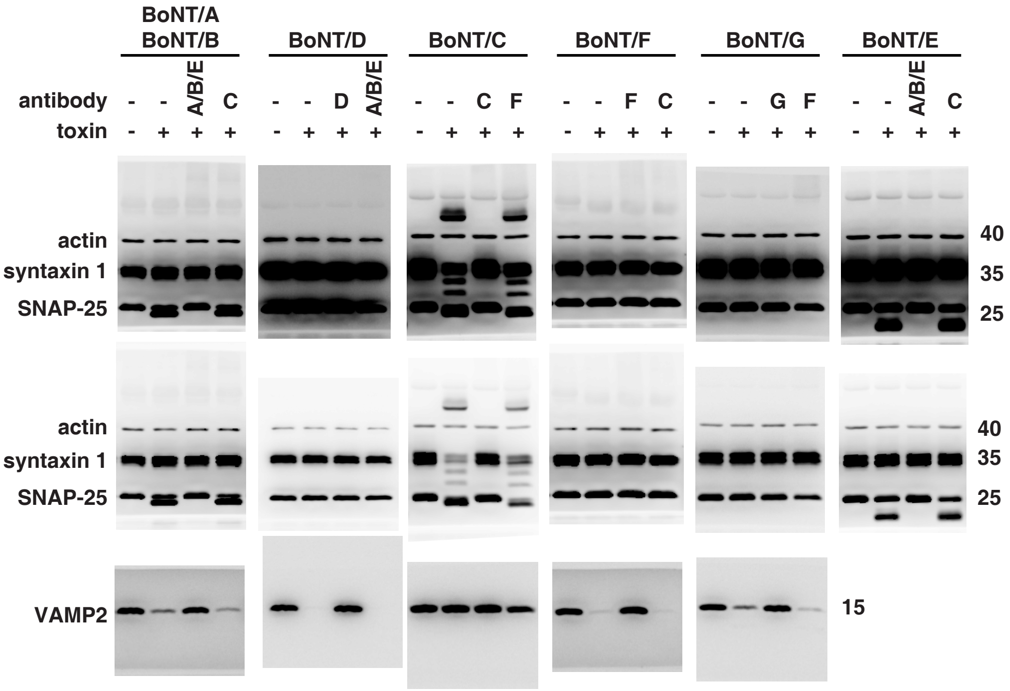

Supplement: Supplementary Information — Supplementary Figures [file ncomms14130-s1.pdf]
